# Assessing the Influence of Zeolite Composition on Oxygen-Bridged Diamino Dicopper(II) Complexes in Cu-CHA DeNO_*x*_ Catalysts by Machine Learning-Assisted X-ray Absorption Spectroscopy

**DOI:** 10.1021/acs.jpclett.2c01107

**Published:** 2022-06-28

**Authors:** Andrea Martini, Chiara Negri, Luca Bugarin, Gabriele Deplano, Reza K. Abasabadi, Kirill A. Lomachenko, Ton V. W. Janssens, Silvia Bordiga, Gloria Berlier, Elisa Borfecchia

**Affiliations:** † Department of Chemistry and NIS Centre, University of Turin, Via Giuria 7, 10125 Turin, Italy; ‡ European Synchrotron Radiation Facility, 71 Avenue des Martyrs, CS 40220, 38043 Grenoble Cedex 9, France; § Umicore Denmark ApS, Kogle Allé 1, DK-2970 Hørsholm, Denmark

## Abstract

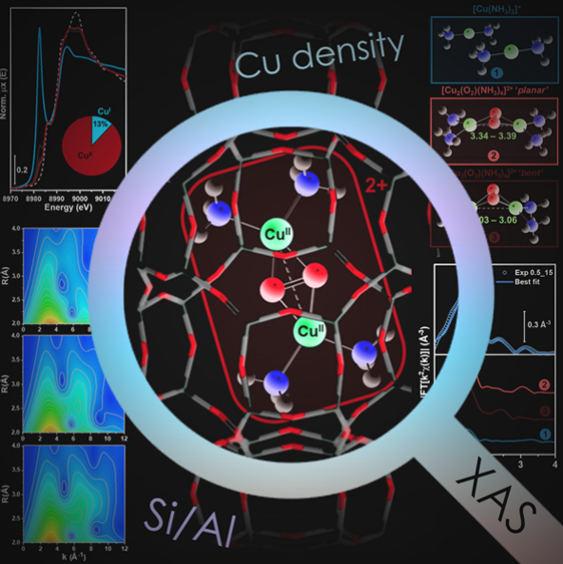

Cu-exchanged chabazite is the catalyst of choice for NO_*x*_ abatement in diesel vehicles aftertreatment systems via ammonia-assisted selective catalytic reduction (NH_3_–SCR). Herein, we exploit *in situ* X-ray absorption spectroscopy powered by wavelet transform analysis and machine learning-assisted fitting to assess the impact of the zeolite composition on NH_3_-mobilized Cu-complexes formed during the reduction and oxidation half-cycles in NH_3_–SCR at 200 °C. Comparatively analyzing well-characterized Cu-CHA catalysts, we show that the Si/Al ratio of the zeolite host affects the structure of mobile dicopper(II) complexes formed during the oxidation of the [Cu^I^(NH_3_)_2_]^+^ complexes by O_2_. Al-rich zeolites promote a planar coordination motif with longer Cu–Cu interatomic distances, while at higher Si/Al values, a bent motif with shorter internuclear separations is also observed. This is paralleled by a more efficient oxidation at a given volumetric Cu density at lower Si/Al, beneficial for the NO_*x*_ conversion under NH_3_–SCR conditions at 200 °C.

Cu-exchanged chabazite (Cu-CHA) currently is the catalyst of choice for the ammonia-assisted selective catalytic reduction (NH_3_-SCR) of harmful NO_*x*_ in diesel vehicles aftertreatment systems.^1−3^ The NH_3_-SCR technology relies on the reaction of NO and NH_3_ in the presence of O_2_, to form N_2_ and H_2_O as benign products, according to the overall stoichiometry 4NH_3_ + 4NO + O_2_ → 4 N_2_ + 6 H_2_O.

Over the past decade, pervasive research efforts have led to a renewed mechanistic understanding of NH_3_–SCR over Cu-CHA, unveiling the atomic-scale structure of the main Cu-species involved.^4−10^ The reaction proceeds via Cu^II^ to Cu^I^ reduction, with release of the products. The catalytic cycle is then closed by reoxidation of Cu^I^ to Cu^II^, requiring the activation of O_2_. The redox-active Cu ions in Cu-CHA are also known to dynamically respond to the gaseous feed across the relevant temperature window, switching between framework-coordinated and mobile configurations.^2,11^

After heating a Cu-CHA catalyst at temperatures above 400 °C and in the presence of O_2_, the Cu is present as a Cu^II^ species, docked to the lattice oxygen atoms in correspondence of the Al exchange sites (Z) in the six- and eight-membered rings (*6r* and *8r*) of the CHA zeolite. Assuming a random Al distribution in the zeolite framework, the chemical identity and local structure are determined by the catalyst composition: low Si/Al and Cu/Al ratios favor bare Z_2_Cu^II^ species in *6r*, while at higher Si/Al and Cu/Al ratios an extra-ligand is required for charge compensation, resulting in Z[Cu^II^OH] or oxygen-bridged multimeric Z_*x*_[Cu_*x*_^II^O_*y*_] species preferentially hosted in *8r*.^12−14^ It is worthwhile to note that deviations from the random Al distribution, known to be promoted by specific synthesis approaches,^15^ could additionally influence the Cu-speciation in dehydrated Cu-CHA. Nonetheless, previous studies^13,14,16^ of the catalysts investigated herein point to a substantial agreement of Cu-speciation in the dehydrated state with the theoretical compositional phase diagram by Paolucci et al.,^12^ based on a random Al distribution in the zeolite framework subject to the Löwenstein’s rule.^17^

At 200 °C, the NH_3_-SCR reaction proceeds in a quasi-homogeneous fashion, over NH_3_-mobilized Cu-ions, yet electrostatically tethered to their framework exchange sites.^6,18^ Spectroscopic studies have been often performed by decoupling the reaction in the reduction and oxidation half-cycles, to facilitate the results interpretation. In the reduction half cycle exposure to a NO/NH_3_ mixture, results in the reduction of all Cu ions to form [Cu^I^(NH_3_)_2_]^+^ complexes. In the oxidation half-cycle, these [Cu^I^(NH_3_)_2_]^+^ complexes are exposed to O_2_, and the interaction of an O_2_ molecule with a pair of [Cu^I^(NH_3_)_2_]^+^ leads to the formation of [Cu_2_(NH_3_)_4_O_2_]^2+^ complexes, which can be described as a *μ-η*^*2*^*,η*^*2*^-peroxo diamino dicopper(II).^19−21^

Paolucci et al. identified the volumetric Cu density as the key descriptor for the efficiency of the oxidation half cycle, validating that O_2_ activation requires a [Cu^I^(NH_3_)_2_]^+^ pair, leading to a quadratic dependency of the NH_3_-SCR rate on Cu density at low Cu-loadings.^6^ Because the mobility of a [Cu^I^(NH_3_)_2_]^+^ complex is limited to distances below approximately 9 Å from their anchoring point in the zeolite, a low Cu density implies a lower propensity for [Cu^I^(NH_3_)_2_]^+^ pair formation, and a larger fraction of unreacted [Cu^I^(NH_3_)_2_]^+^. As the O_2_ activation implies a change in the oxidation state of Cu^I^ to Cu^II^, the fraction of unreacted [Cu^I^(NH_3_)_2_]^+^ can be readily quantifiable by Cu K-edge X-ray Absorption Spectroscopy (XAS).

In this scenario, the actual reaction takes place on mobile Cu species, and therefore, the stable position of framework-bound Cu ions seems less relevant for the NH_3_-SCR activity. However, the density and distribution of the framework Al atoms and Brønsted acid sites may influence the mobility of the [Cu^I^(NH_3_)_2_]^+^ complexes, the stability of Cu pairs, and thus the formation of the [Cu_2_(NH_3_)_4_O_2_]^2+^ complexes. The potential implications for low-temperature NH_3_-SCR performance through such zeolite-host driven changes in [Cu_2_(NH_3_)_4_O_2_]^2+^ formation are still unexplored.

To determine the influence of the zeolite properties on the NH_3_-SCR reaction, we performed an *in situ* XAS study empowered by wavelet transform (WT) analysis and machine learning (ML)-assisted fitting of the extended X-ray absorption fine structure (EXAFS) spectra in order to interrogate three Cu-CHA catalysts with variable Si/Al ratios and Cu contents during the reduction and oxidation half-cycles in NH_3_-SCR at 200 °C. Cu content and Si/Al ratio in the catalysts are balanced in such a way that the Cu density in two of the three catalysts is equivalent, despite having different Si/Al ratios. The catalysts used in this study have a Si/Al ratio of 5, 15 and 29, with a Cu/Al ratio of 0.1, 0.5, and 0.6 (estimated Cu densities of 0.28, 0.44, and 0.28 Cu/Å^3^, respectively), denoted hereafter as “0.1_5”, “0.5_15”, and “0.6_29”. These catalysts have been characterized previously in depth after pretreatment in O_2_ at 400 °C.^14^ We use the 0.5_15 catalyst as a reference, because it was previously investigated under the same redox conditions.^20^ Additional details on the investigated catalysts, as well as on the experimental methods for *in situ* XAS data collection can be found in the SI, Section 1.

Figure 1a–c compares the Cu K-edge X-ray absorption near edge structure (XANES) spectra of the three catalysts measured after pretreatment in O_2_ at 400 °C and cooling down to 200 °C in the same gas feed, at the end of the reduction step in NO/NH_3_ and during the exposure of the reduced catalyst to O_2_, also collected under isothermal conditions at 200 °C. In parallel, Figure 1d–f illustrates the main envisaged Cu-species at each step in this process.

**Figure 1:**
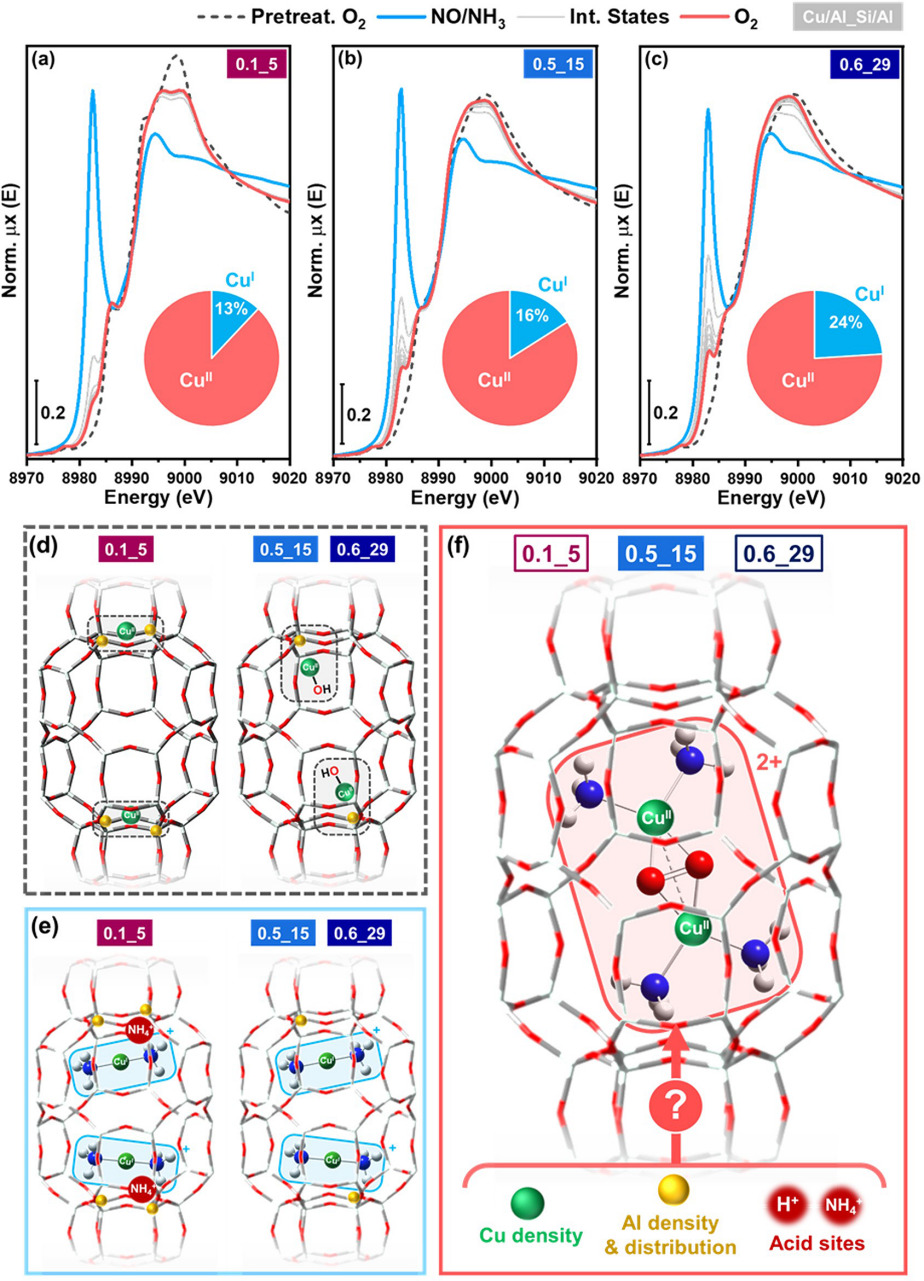
*In situ* Cu K-edge XANES for (a) 0.1_5, (b) 0.5_15, and (c) 0.6_29 Cu-CHA catalysts collected at 200 °C after pretreatment in O_2_, at the end of the reduction step, during and after the oxidation step. Pie charts illustrate Cu^I^/Cu^II^ percentages evaluated by XANES LCF at the end of the oxidation step for each catalyst; under the adopted experimental conditions, Cu^I^ and Cu^II^ correspond to [Cu^I^(NH_3_)_2_]^+^ and [Cu_2_(NH_3_)_4_O_2_]^2+^ complexes, respectively. (d–f) Pictorial representation of the main Cu-species expected at each step as a function of the catalyst composition, with open questions on parameters influencing coordination motif in [Cu_2_(NH_3_)_4_O_2_]^2+^ complexes in part (f).

For all three catalysts, the XANES after pretreatment in O_2_ at 400 °C (dashed gray curves in Figure 1) shows the expected features for Z_2_Cu^II^ and Z[Cu^II^OH]/Z_*x*_[Cu_*x*_^II^O_*y*_] species, reflecting their different Cu/Al and Si/Al ratios.^12−14^ At the end of the reduction step, for each catalyst, the XANES indicates the formation of mobile [Cu^I^(NH_3_)_2_]^+^ for all the Cu ions present. For the 0.1_5 composition, the reduction to [Cu^I^(NH_3_)_2_]^+^ of the dominant Z_2_Cu^II^ species implies the formation of a new Brønsted acid site to maintain the charge balance in the zeolite. As NH_3_ is present in the applied reduction conditions, this results in the formation of NH_4_^+^ ions (Figure 1e).

During the oxidation step, the characteristic XANES features of Cu^II^ progressively develop, in line with the formation of [Cu_2_(NH_3_)_4_O_2_]^2+^ complexes.^6,20^ However, the spectral shape reached at the end of the oxidation step for the 0.1_5 catalyst differs from those observed for the 0.5_15 and 0.6_29 catalysts, especially in the white-line peak region. This indicates a different coordination motif for [Cu_2_(NH_3_)_4_O_2_]^2+^ in the Al-rich 0.1_5 catalyst, and therefore, we undertook a more in-depth analysis of the *in situ* XAS data.

Although the majority of the Cu ions are reoxidized to Cu^II^ after the oxidation step, from XANES linear combination fit (LCF) we found a residual amount of [Cu^I^(NH_3_)_2_]^+^, determined by CHA Si/Al ratio, and ranging from 13% for Si/Al = 5, to 16% for Si/Al = 15 and 24% for Si/Al = 29 (see pie charts in Figure 1a-c and SI, Section 2 for details).

Figure 2a reports the NO_*x*_ conversion in NH_3_-SCR for the three catalysts as a function of temperature. At 200 °C, 0.1_5 and 0.5_15 show almost equivalent conversion, which is instead lower for 0.6_29. The inset of Figure 2a correlates the turnover frequency (TOF), obtained by converting the measured NO_*x*_ conversion at 200 °C to a rate constant based on a first order kinetic model, corresponding to an integral reactor analysis,^22^ and accounting for Cu wt % in the catalysts (SI, Table S1). Considering the increase in residual Cu^I^ as Si/Al increases observed from XANES LCF, the TOF at 200 °C follows the opposite trend: it is higher in 0.1_5 and it decreases in an approximately linear way for the 0.5_15 and 0.6_29 catalysts. This indicates that a more efficient oxidation of [Cu^I^(NH_3_)_2_]^+^ by O_2_ activation is beneficial for the low-temperature NH_3_-SCR performance.

**Figure 2:**
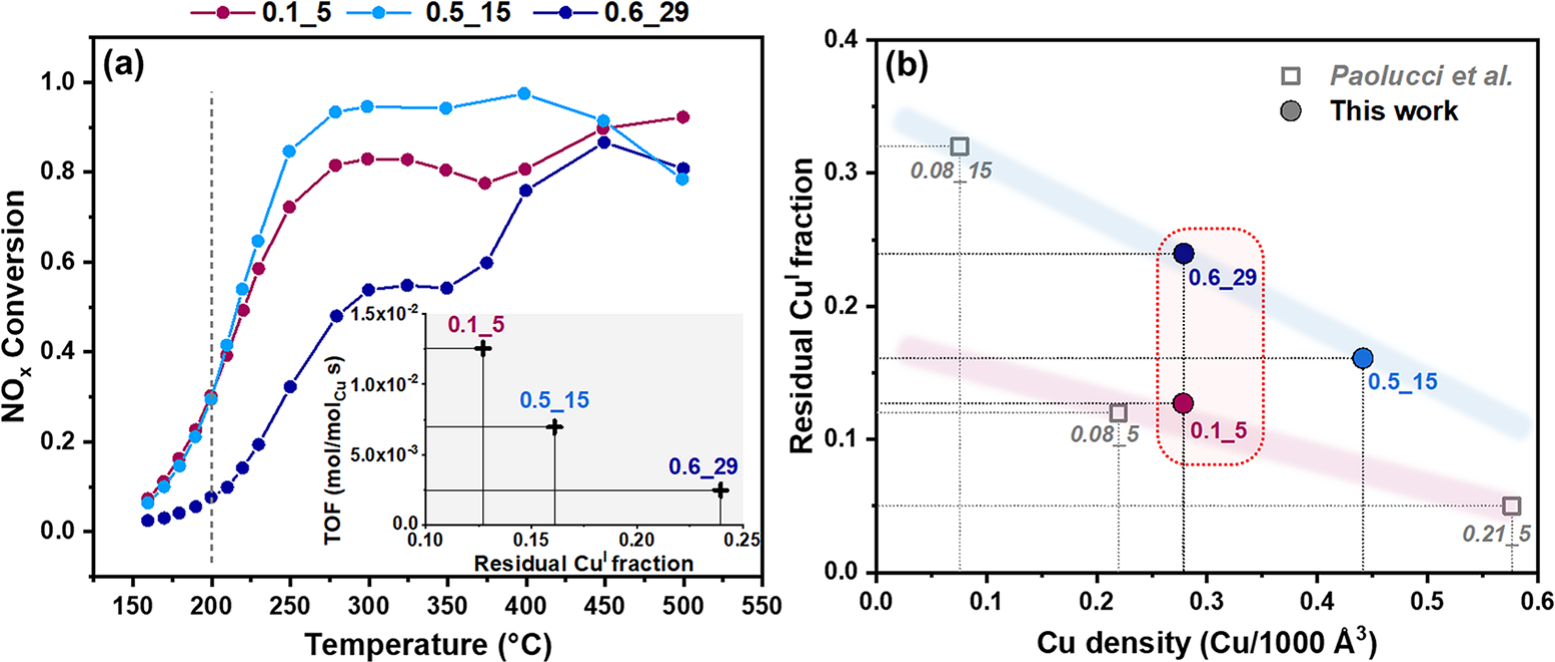
(a) NO_*x*_ conversion in the 150–500 °C temperature range for 0.1_5, 0.5_15, and 0.6_29. Amount of catalyst: 5 mg; feed gas: 500 ppm of NO, 533 ppm of NH_3_, 5% H_2_O, 10% O_2_ in N_2_; flow: 225 N mL/min. The bottom inset correlates residual Cu^I^ fractions evaluated by XANES LCF and turnover frequency (TOF) in mol/[mol_Cu_ s] at 200 °C for the three catalysts. (b) Correlation between the same residual Cu^I^ fractions and the volumetric Cu density in Cu-CHA catalysts, comparing experimental values obtained in this work (colored full circles) and literature values by Paolucci et al.^6^ (empty gray squares).

We note that the difference in residual Cu^I^ fractions is not entirely determined by the Cu density, but that the Si/Al ratio also plays a role. Figure 2b compares Cu density with the residual Cu^I^ fraction for the three catalysts investigated in this study and for the data by Paolucci et al.^6^ It is worth noticing that the 0.1_5 and 0.6_29 catalysts show comparable Cu density, while the residual Cu^I^ fraction after oxidation is significantly lower for 0.1_5 than for 0.6_29. The data appear to branch based on the Si/Al ratio, with a different dependence on the Cu density for low and high Si/Al ratios (purple and blue shadowed areas in Figure 2b, for Si/Al = 5 and Si/Al = 15, 29, respectively). Overall, at comparable Cu density, for Si/Al = 5 a lower residual Cu^I^ fraction is observed than for higher Si/Al ratios. This behavior is reminiscent of the compositional effects on Cu-speciation after pretreatment in O_2_, when low Si/Al favors Z_2_Cu^II^ at the expense of Z[Cu^II^OH],^12,13^ although the oxidation step proceeds from mobile [Cu^I^(NH_3_)_2_]^+^, spectroscopically indistinguishable over the compositional series.

To correlate quantitatively the structural differences in the CHA zeolites with the observed trends in oxidation efficiency, we compared the EXAFS of the three catalysts at the end of both the reduction and oxidation steps (Figure 3). Together with the conventional Fourier transform (FT)-EXAFS representation, we exploited a WT-based analysis^23,24^ to unambiguously identify possible Cu–Cu scattering contributions possessing a characteristic lobe centered at ca. 7 Å^–1^ along the *k* direction^14,20,25−28^ (SI, Section 3 for details).

**Figure 3:**
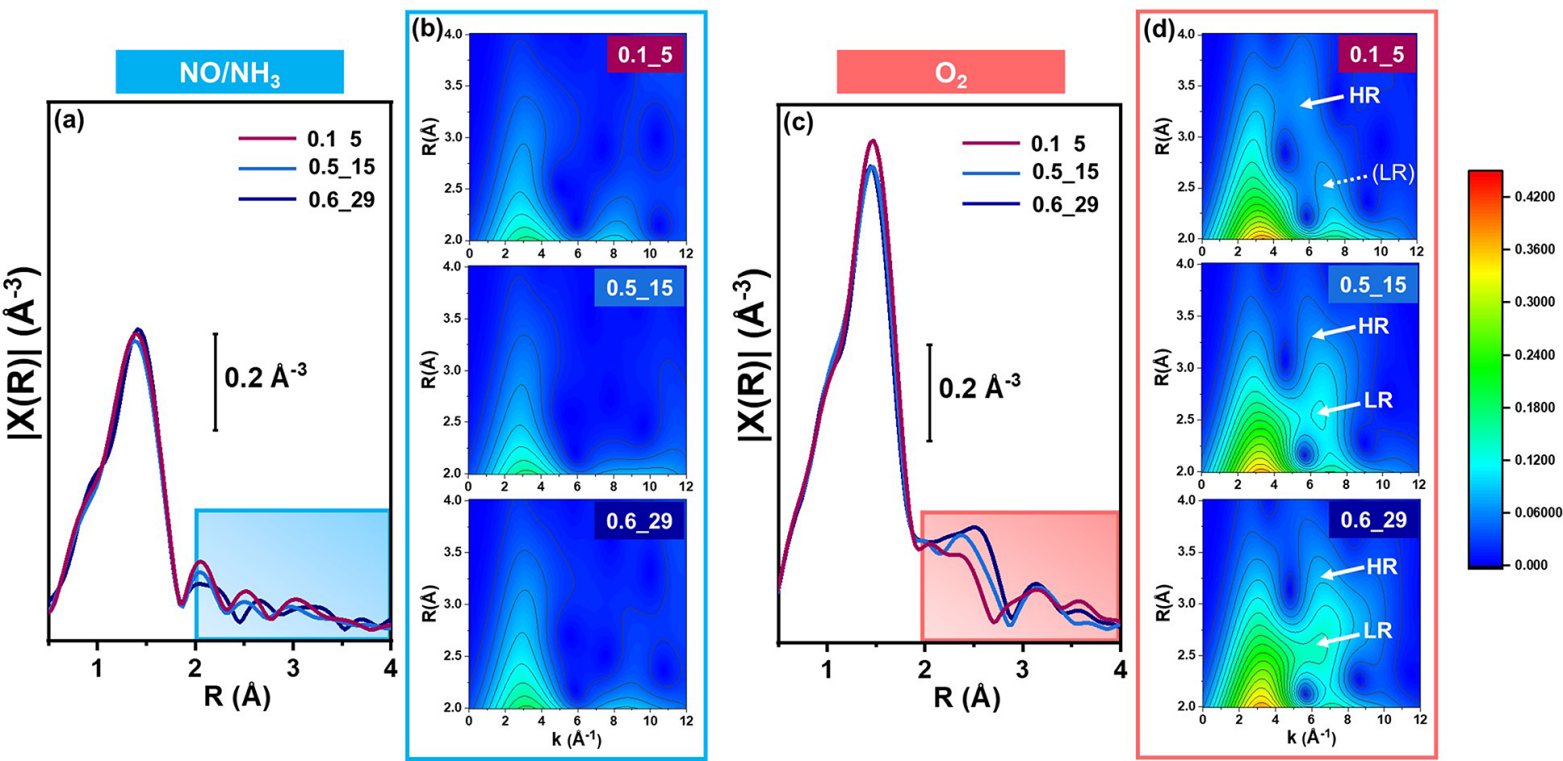
Snapshots of local coordination environment of Cu ions by EXAFS, comparing 0.1_5, 0.5_15 and 0.6_29 at the end of the (a, b) reduction and (c, d) oxidation steps. (a, c) Magnitude of FT-EXAFS spectra, obtained by Fourier transforming *k*^2^χ(*k*) spectra in the 2.4–12.0 Å^–1^ range. (b, d) Corresponding EXAFS-WT maps, magnified in the 2–4 Å range and plotted using a common intensity scale. Low-R (**LR**) and high-R (**HR**) features in the *k*-range diagnostic of Cu–Cu scattering are highlighted by white arrows in part (d).

EXAFS confirms that after the reduction step all the Cu is present as [Cu^I^(NH_3_)_2_]^+^ complexes, without any detectable dependence on the Cu and Al contents in the catalyst (Figure 3a,b). In all cases, unstructured FT-EXAFS features are observed beyond the first-shell peak originating from Cu–N single scattering. In parallel, the WT maps only shows a low-k lobe assigned to multiple scattering paths involving the two N atoms of the NH_3_ ligands. EXAFS fits based on the [Cu^I^(NH_3_)_2_]^+^ structure confirmed this picture (SI, Section 4.2).

In all cases, the FT-EXAFS shows a first-shell peak with the same intensity at the end of the oxidation step, indicating 4-fold coordinated Cu^II^ ions. In the *R*-range within 2–4 Å, a broad peak centered at 2.5 Å grows in intensity in the order 0.1_5 < 0.5_15 < 0.6_29, accompanied by further variations in the 3–4 Å range (Figure 3c). The WT maps show a low-*k* lobe originating from multiple scattering contributions from low-Z (O and N) neighbors for all catalysts (see Figure 3d and SI, Figure S5b for the corresponding *Φ*^*R*^(*k*)density power functions), with an intensity and R-space location that is only slightly affected by the catalyst composition. In the high-*k* range characteristic of Cu–Cu scattering, we observe two local maxima along the *R* direction at ca. 2.5 and 3.2 Å (**LR** and **HR** labels in Figure 3d, respectively), pointing to an EXAFS-resolvable bimodal distribution of Cu–Cu interatomic distances. The **LR** feature becomes more intense relative to the **HR** feature as the Si/Al ratio increases; the **HR** feature is also inherently weaker due to the dampening of the EXAFS signal as R increases.

Taking into account phase correction, the **HR** feature is compatible with the DFT-optimized geometry of quasi-planar *μ-η*^*2*^*,η*^*2*^-peroxo diamino dicopper(II) with intranuclear separation of 3.40 Å, as reported for 0.5_15 in our previous work.^20^ However, the conventional EXAFS refinement solely based on this model revealed a local lack of fit at ca. 2.5 Å (SI, Section 4.3). This corresponds to the **LR** feature becoming more visible for 0.6_29 in the WT maps.

To further understand the emerging structural complexity, we employed a novel ML-assisted EXAFS analysis approach^29−31^ to build up a robust three-component fitting model. The model accounts for a minor presence of [Cu^I^(NH_3_)_2_]^+^ (Figure 4a, **1**) and for a bimodal distribution of the Cu–Cu distances in *μ–η*^*2*^*,η*^*2*^-peroxo diamino dicopper(II). On the basis of the WT analysis, the latter is described by combining contributions from the already proposed quasi-planar configuration (Figure 4a, **2**/**2′**) and a “bent” motif (Figure 4a, **3**) for the same complex, refined through ML-based optimization of the Cu–O–Cu angle (see SI, Section 5 for a detailed description of the methodology and a complete report on fitting results).

**Figure 4:**
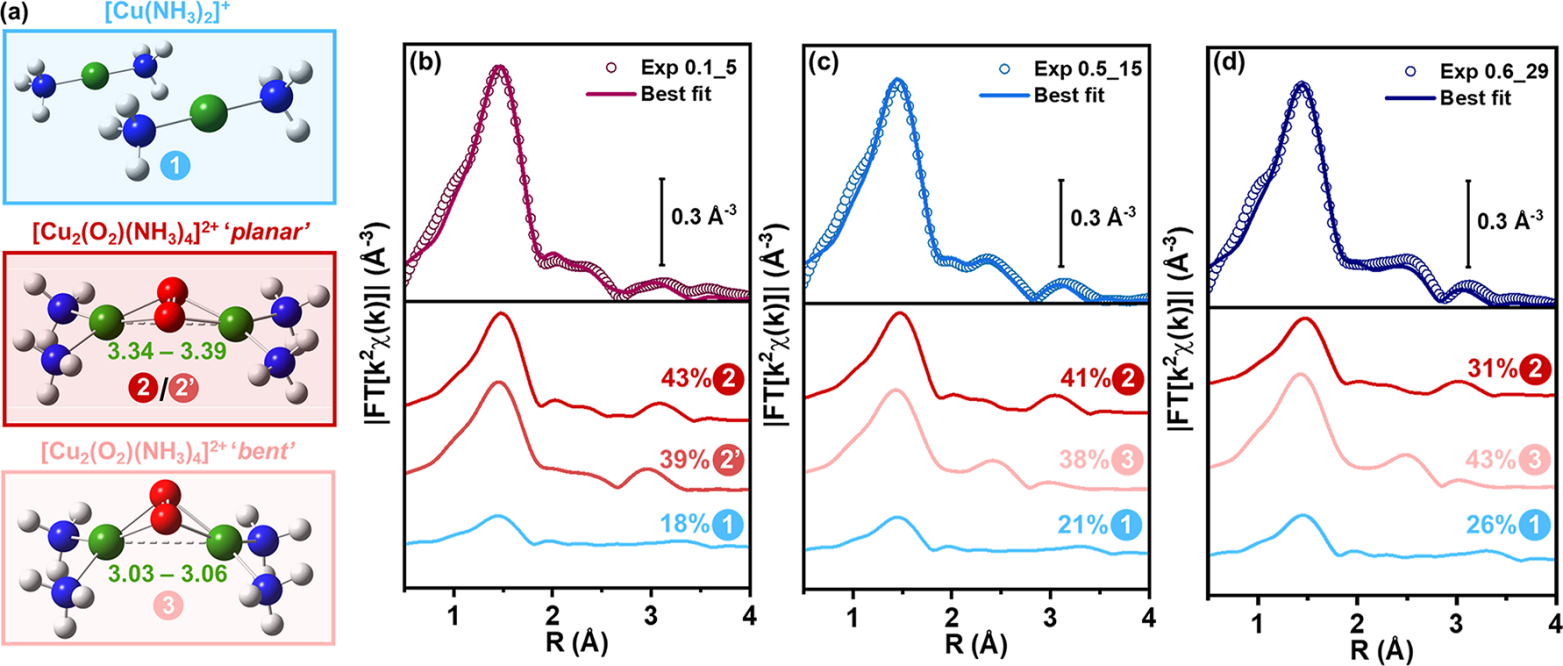
(a) Molecular models for the structural components included in the Machine Learning (ML)-assisted EXAFS fitting model: **1** [Cu^I^(NH_3_)_2_]^+^, **2** “planar”, and **3** “bent” motifs for *μ-η*^*2*^*,η*^*2*^-peroxo diamino dicopper(II); when relevant, characteristic EXAFS-derived ranges for Cu–Cu interatomic distances are reported, in Å. (b–d) Comparison between magnitudes of experimental (colored circles) and best fit (thick lines) FT-EXAFS spectra at the end of the oxidation step for (b) 0.1_5, (c) 0.5_15, and (d) 0.6_29 (see SI, Figure S13 for the corresponding imaginary parts). The scaled components for Cu-species **1**, **2**/**2′**, **3** are also reported, vertically translated for the sake of clarity, together with percentages of each component over total Cu refined by ML-EXAFS fitting.

Figure 4b–d compares the corresponding experimental and best-fit EXAFS spectra for the three catalysts, highlighting the individual scattering contributions and refined percentage for each Cu-species. The percentages of residual [Cu^I^(NH_3_)_2_]^+^ agree well with those from XANES LCF, revealing the same trend in the oxidation efficiency as a function of the Si/Al ratio. The previously observed lack of fit at ca. 2.5 Å is now resolved for the whole set of samples.

The fit confirms that two distinct *μ-η*^*2*^*,η*^*2*^-peroxo diamino dicopper(II) coordination motifs are required to fully model the EXAFS of 0.5_15 and 0.6_29, which is reflected in a different Cu–Cu distance for the bent (3.03–3.06 Å) and planar complex (3.34–3.39 Å). Interestingly, for 0.1_5 the three-component fit restitutes Cu–Cu distances of 3.38 and 3.39 Å, not distinguishable within the accuracy of EXAFS analysis and both pointing to the same planar motif (**2**/**2′** labels in Figure 4b). The bent motif is instead progressively favored as the Si/Al ratio increases, becoming the major structural component for the 0.6_29 sample.

ML-EXAFS fitting ensures a quantitative understanding of the subtle differences observed in the XANES spectral shape as well as in the high-R portion of WT maps, where the **LR**/**HR** features fairly match the optimized Cu–Cu distances for planar and bent *μ-η*^*2*^*,η*^*2*^-peroxo diamino dicopper(II). The adopted approach allows refining, in principle, any structure starting from an initially guessed molecular complex with significantly different interatomic distances and angles. These results therefore highlight the potential of *in situ* XAS combined with integrated WT and ML-assisted EXAFS analysis.

Summarizing, the XANES and EXAFS results for the Cu-CHA catalysts consistently show, that the Si/Al ratio of the zeolite host affects the structure of mobile dicopper(II) complexes formed during the oxidation of the [Cu^I^(NH_3_)_2_]^+^ complexes by O_2._ Diffuse Reflectance UV–vis spectroscopy, qualitatively responsive to the structure of Cu-oxo species, further supports these results (see SI, Section 6). Therefore, we suggest that the diverse electrostatic landscape and the higher Brønsted acid site density in Al-rich zeolites could trigger host–guest interactions promoting a longer internuclear separation in the planar *μ-η*^*2*^*,η*^*2*^-peroxo diamino dicopper(II) motif. Plausibly, the NH_3_ ligands in the dicopper(II) complex could participate into H-bonding interactions with ZH sites, as well as ZNH_4_ and ZNH_4_*n*NH_3_ associations, most likely formed at the reduction step in the presence of NH_3_ at 200 °C,^32^ triggering the observed elongation effect in Cu–Cu distances. Importantly, this is accompanied by a more efficient oxidation at a given volumetric Cu density, which is beneficial for the NO_*x*_ conversion under NH_3_-SCR conditions at 200 °C. With this respect, Al-rich zeolites could favor dynamic Cu ion exchange between nearby sites, thus enhancing the mobility of [Cu^I^(NH_3_)_2_]^+^ complexes beyond the limit dictated by electrostatic tethering to the initial exchange sites. A similar mechanism based on H^+^/H_2_O-aided diffusion of Cu^I^ was proposed in the context of continuous partial oxidation of methane to methanol over Cu-CHA, although in this case ZNH_4_ is suggested to hinder the exchange pathway.^33^ Overall, the findings obtained in this work provide a robust experimental basis for further theoretical modeling of low-temperature NH_3_-SCR mechanism, aimed at validating these hypotheses within a quasi-homogeneous Cu-catalyzed oxidation half-cycle actively driven by the compositional characteristics of the zeolitic host.

## Supporting Information Available

Details on compositional characteristics, evaluation of Cu density and synthesis methods for the investigated Cu-CHA catalysts. Experimental methods for *in situ* XAS and UV–vis spectroscopies. Additional information about methods, models and supplementary results for the employed XAS data analysis strategies: XANES LCF, EXAFS WT, conventional EXAFS fitting, and ML-assisted EXAFS fitting. Complementary *in situ* UV–vis spectroscopy results (PDF)
