# Supplementary material for: Assessing the Influence of Zeolite Composition on Oxygen-Bridged Diamino Dicopper(II) Complexes in Cu-CHA DeNOx Catalysts by Machine Learning-Assisted X-ray Absorption Spectroscopy
Source: J Phys Chem Lett. 2022 Jun 28;13(26):6164–70. doi: 10.1021/acs.jpclett.2c01107 (PMC9272442; doi:10.1021/acs.jpclett.2c01107)
Supplement: Supplementary file 1 — jz2c01107_si_001.pdf [file jz2c01107_si_001.pdf]

Supporting Information

Assessing the Influence of Zeolite Composition on Oxygen-Bridged Diamino Dicopper(II) Complexes in Cu-CHA $DeNO_x$ Catalysts by Machine Learning-Assisted X-ray Absorption Spectroscopy

*Andrea Martini,[a,1] Chiara Negri,[a,2] Luca Bugarin,[a,b] Gabriele Deplano,[a] Reza K. Abasabadi,[a] Kirill A. Lomachenko,[b] Ton V. W. Janssens,[c] Silvia Bordiga,[a] Gloria Berlier,[a] Elisa Borfecchia,[a]**

[a] Department of Chemistry and NIS Centre, University of Turin, Via Giuria 7, 10125 Turin, Italy

[b] European Synchrotron Radiation Facility, 71 Avenue des Martyrs, 38043 Grenoble Cedex 9, France

[c] Umicore Denmark ApS, Kogle Allé 1, DK-2970 Hørsholm, Denmark

Corresponding Author

*elisa.borfecchia@unito.it

[1] Present address: Interface Science Department, Fritz Haber Institute, Faradayweg 4-6, 14195 Berlin, Germany

[2] Present address: Politecnico di Milano, Laboratory of Catalysis and Catalytic Processes, Department of Energy, via La Masa, 34, 20156 Milano, Italy

Table of Contents

1 Materials and Methods 3

1.1 Investigated Cu-CHA catalysts: compositional characteristics and evaluation of Cu density 3

1.2 In situ XAS experiments 4

1.3 In situ DR UV-Vis experiments 5

2 Residual Cu(I) fraction during the oxidation step estimated by XANES LCF analysis 6

3 Wavelet Transform (WT) EXAFS analysis after the reduction and oxidation steps 9

3.1 Methodological background 9

3.2 Full-range WT-EXAFS maps after reduction and oxidation steps 12

3.3 Density power functions after reduction and oxidation steps 12

4 Conventional EXAFS fitting results 13

4.1 Methods and adopted models for conventional EXAFS fitting 13

4.2 Conventional EXAFS fitting results after the reduction step 14

4.3 Preliminary results from conventional EXAFS fitting after the oxidation step for 0.5_15 and 0.6_29 16

5 EXAFS fitting after the oxidation step through a FEFF-based Machine Learning Indirect Approach ... 18

5.1 Methodological background 18

5.2 Fitting model and parametrization strategy 20

5.3 Detailed report on fitting results 22

6 DR-UV-Vis spectra 27

7 References 29

1 Materials and Methods

1.1 Investigated Cu-CHA catalysts: compositional characteristics and evaluation of Cu density

The three Cu-CHA catalysts investigated in this work are denoted with "Cu/Al_Si/Al" labels, namely 0.1_5, 0.6_29 and 0.5_15 respectively. Detailed information on the synthesis procedures can be found in previously published works.[1, 2] **Table S1** reports an overview on the compositional characteristics of the three samples (determined by ICP-OES) together with their respective Al (N_{Al}) and Cu (N_{Cu}) densities and mean Cu-Cu interatomic distances inside the CHA framework, calculated as detailed below.

Table S1. Compositional parameters, Al and Cu density inside the CHA framework and mean Cu-Cu distance of the 0.1_5, 0.5_15 and 0.6_29 Cu-CHA samples analyzed via *in situ* XAS.

Cu-CHA sample	Si/Al	Cu/Al	Cu wt.%	N_{Al} (Al/1000 Å^3)	N_{Cu} (Cu/1000 Å^3)	Mean Cu-Cu distance (Å)
**0.1_5**	4.94	0.11	1.51	2.54	0.28	18.98
**0.5_15**	15.12	0.47	2.64	0.94	0.44	16.30
**0.6_29**	29.17	0.56	1.71	0.50	0.28	19.00

N_{Al} and N_{Cu} were calculated as follows:

$$N_{Al} = \frac{FD_{Si}}{(1 + N_{Si}/N_{Al})} \qquad \text{(S1.1)}$$

$$N_{Cu} = \frac{N_{Cu}}{N_{Al}} \times N_{Al} \qquad \text{(S1.2)}$$

Where FD_{Si} is the Si framework density inside the CHA zeolite (15.1 T/1000 Å^3 and N_{Si}/N_{Al} and N_{Cu}/N_{Al} are the Si/Al and Cu/Al ratios characterizing the different Cu-CHA samples reported in **Table S1**.

The mean Cu-Cu distances were estimated assuming that the Cu atoms are homogeneously distributed and that each Cu can be considered as a sphere with a radius R. Thus, the Cu sphere volume ($V_{\mathrm{Cu-sphere}}$) was defined as:

$$V_{\mathrm{Cu-sphere}} = \frac{4}{3} \times \pi \times R^3 \qquad \text{(S1.3)}$$

This volume was assumed to be same as the $Volume\ per\ Cu$:

$$V_{\mathrm{Cu-sphere}} = Volume\ per\ Cu = \frac{V_{CHA}}{n_{Cu}} \qquad \text{(S1.4)}$$

The Cu density previously calculated can be also estimated as:

$$N_{Cu} = n_{Cu} \times \frac{1000}{V_{CHA}} \qquad \text{(S1.5)}$$

Where V_{CHA} is the volume of the CHA zeolite cell (2391.5 Å^3) and n_{Cu} is the number of Cu atoms for CHA unit cell. Substituting n_{Cu} from eq. (S1.5) into eq. (S1.4), we finally obtain $V_{\text{Cu-sphere}}$ as:

$$V_{\text{Cu-sphere}} = \frac{1000}{N_{Cu}} \qquad \text{(S1.6)}$$

Finally, the mean Cu-Cu distances can be found considering two times the radius of the Cu sphere, *R*:

$$\textit{Mean Cu-Cu distance} = 2R = 2 \times \sqrt[3]{\frac{V_{\text{Cu-sphere}}}{\pi} \times \frac{3}{4}} \qquad \text{(S1.7)}$$

According to the Cu-CHA phase diagram by Paolucci *et al.*,[3] as well as previous characterization work[2, 4, 5], the investigated Cu-CHA samples after pretreatment in O_2 are characterized by two main types of framework-coordinated Cu^{II} species. 0.1_5 mostly contains Z_2Cu^{II} at *2Al* sites in 6r, whereas $Z[Cu^{II}OH]$ at *1Al* sites in 8r and structurally related multimeric moieties $Z_x[Cu^{II}_xO_y]$ are largely dominant for 0.5_15 and 0.6_29.

1.2 In situ XAS experiments

X-ray Absorption Spectroscopy (XAS) experiments were performed at the BM23 beamline[6] of the European Synchrotron Radiation Facility (ESRF, Grenoble, France) using the Microtomo reactor cell.[7] The Cu-CHA catalysts were prepared in the form of self-supporting pellets with optimized masses for XAS data collection in transmission mode (100-120 mg) and fixed inside the Microtomo reactor cell for *in situ* experiments.

For all the *in situ* XAS experiments, a total gas flow of 50 ml/min was used. In all cases, the measured catalyst was initially pre-treated in O_2 at 400 °C for 60 min (heating rate 5 °C/min), then cooled to 200 °C in O_2 and exposed to 1000 ppm NO/1000 ppm NH_3/He gas mixture at the same temperature (reduction step). Once steady state conditions were reached (typically after 90 min), the system was purged with He, then exposed to 10% O_2/He (oxidation step) and monitored for ca. 40 min, until a substantial stabilization of the spectroscopic features was observed. All the *in situ* XAS data reported in this work were collected at 200 °C.

Cu K-edge XAS spectra were collected in transmission mode. A double-crystal Si(111) monochromator was employed for the incident energy scan, with a pair of flat Si mirrors at 2.5 mrad angle for harmonics rejection. Two ionization chambers allowed detecting incident (I_0) and transmitted ($I_{1,2}$) photons, while a third ionization chamber (I_2) was employed to measure simultaneously the XAS spectrum of a Cu metal foil, for the sake of

an accurate energy calibration and alignment of the *in situ* XAS data. Cu K-edge XAS spectra were collected in the 8800 – 9965 eV energy range (pre-edge region energy step = 5 eV, edge region energy step = 0.3 eV, constant k-space sampling $\Delta k = 0.035$ Å^{-1} in the EXAFS region; acquisition time of 1 s/point).

XAS data treatment, including energy calibration/alignment, normalization, extraction of the $\chi(k)$ functions and calculation of Fourier transform of the $k^2\chi(k)$ functions (2.4 – 12.0 Å^{-1} k-range) was performed using the Athena software from the Demeter package.[8]

Detailed descriptions of the methods adopted for XANES linear combination fit (LCF) analysis, EXAFS Wavelet Transform (WT) analysis and Machine Learning (ML)-assisted fitting can be found separately in the following Sections.

1.3 In situ DR UV-Vis experiments

Diffuse Reflectance (DR) UV-Vis measurements have been performed with an Avantes AvaSpec-ULS2048XL-EVO fiber optics spectrometer (100µm slits), coupled to an Avantes AvaLight-DH-S light source (equipped with a deuterium and a halogen lamp) with an integration time of 10 ms and averaging 50 scans. A custom-designed high-temperature resistant fiber optic with a high-OH fused silica core of 100 µm in diameter was used for the collection of diffuse reflected light from the sample. Polytetrafluoroethylene (PTFE) powder was used as a standard for 100% reflectance. All measurements were performed in a sapphire tubular reactor of 2 mm internal diameter and 10 cm long. Heating was achieved with a Ni-Cr coil (approximately 12 Ω resistance) powered by a low-voltage power supply controlled through a PID. Spectra are reported as relative reflectance (R%) defined as:

$$R\% = {}^{R_{sample}}\!/_{R_{reference}}\ x\ 100$$

The protocol followed for the *in situ* experiments was similar to what described for *in situ* XAS (Section 1.2).

2 Residual Cu(I) fraction during the oxidation step estimated by XANES LCF analysis

XANES Linear Combination Fit (LCF) analysis is a well-established method for the semi-quantitative determination of the absorber oxidation states/speciation, by reconstructing the experimental spectrum $\mu^{EXP}(E)$ as a linear combination of selected reference spectra $\mu_i^{REF}(E)$, i.e., $\mu^{LCF}(E) = \sum_i w_i \, \mu_i^{REF}(E)$, optimizing the weights w_i for each reference spectrum.

Paolucci *et al.*[9] reported a Cu K-edge XANES LCF analysis on a set of Cu-CHA catalysts monitored under equivalent chemical treatment conditions as adopted in this work for the oxidation step, although with a different experimental setup ensuring higher time resolution and access to kinetically-relevant information. Their analysis shows that for all the samples, the higher is the Cu density inside the framework the higher is the Cu^{I} to Cu^{II} conversion reached at the steady state. On these bases, we decided to apply the same LCF approach on the *in situ* XANES spectra of our Cu-CHA samples during the oxidation step.

To this aim, we selected in the same way the reference spectra for the Cu^{I} and Cu^{II} components. Specifically, for each sample we considered the last XAS spectrum measured after pre-treatment and cooling in O_2 at 200 °C and the last scan measured during the reduction step (NO/NH_3 at 200 °C, safely assigned to $[Cu^{I}(NH_3)_2]^{+}$ complexes) as reference for the Cu^{II} and Cu^{I} species, respectively, according to the LCF model adopted in ref.[9]

LCF was performed using the Athena software from the Demeter suite,[8] in the energy range 8970 − 9020 eV, by imposing the mass balance condition ($\sum_i w_i = 1$) and the non-negativity ($w_i \geq 0, \forall\, w_i$) conditions for the optimized weights w_i.

Figure S1 reports the Cu^{I} fraction evolution over the experimental time for our samples (colored circles) compared with the Cu^{I} 'plateau values' found by Paolucci *et al.*[9] (horizontal solid lines).

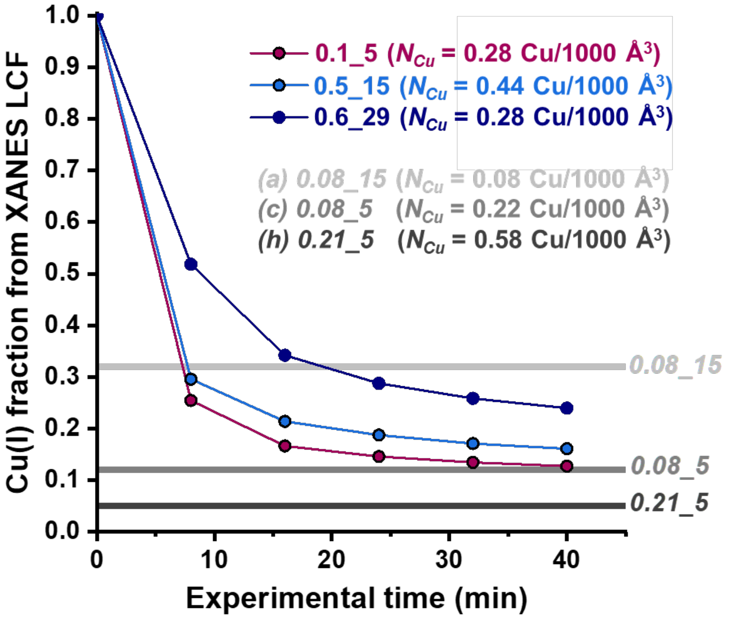

Figure S1: Cu^I fraction evolution vs experimental time obtained from Cu K-edge XANES LCF analysis during the oxidation step at 200 °C for 0.1_5 (purple circles), 0.5_15 (blue circles), and 0.6_29 (dark blue circles) Cu-CHA catalysts compared to the Cu^I 'plateau values' found by Paolucci *et al.*[9] (horizontal light grey, grey and dark grey solid lines, with labels reporting the respective compositional characteristics, using the same notation as employed to denote the samples investigated here). Statistical LCF errors on Cu^I fractions are of ± 3% total Cu. The legend also reports Cu density values for the two series of samples, as well as (a), (c), (h) labels used to identify the catalysts in the original work by Paolucci *et al.*[9]

Notably, we observe a substantial agreement, within the statistical LCF errors (± 3% total Cu in all cases), between the residual Cu(I) fraction estimated for the catalysts with comparable compositional characteristics (Cu/Al and Si/Al ratios), namely 0.1_5 in our series and 0.08_5 in the previous work by Paolucci *et al.* Moreover, considering only the 0.5_15 and 0.6_29 samples, we can observe that they reached different plateau values in the Cu^I residual fraction, in agreement with the interpretative model proposed by Paolucci *et al.* based on Cu-pairs formation: the higher the framework Cu density, the lower the residual fraction of Cu^I. However, samples 0.6_29 and 0.1_5 possess the same Cu density but significantly differs in Cu^I residual fraction, with 0.1_5 being the sample with the lowest residual Cu(I) fraction in our series.

The LCF resulted in a satisfactory reproduction of the experimental XANES spectra, with overall R-factor values < 0.004. For indication, **Figure S2** shows representative LCF curves compared with the corresponding experimental spectra, at the end of the oxidation step for each of the three Cu-CHA catalysts investigated in this work. The reproduction of the experimental spectrum is particularly good in the pre-edge/rising-edge XANES region, especially with respect to the Cu^I fingerprint peak at ca. 8983 eV, while, not surprisingly, a certain degree of misfit is found in the white-line region. This issue

directly stems from the Cu^{II} reference spectra chosen to perform the LCF (according to the same criteria employed in ref.[9]) that is faithfully associated to a Cu^{II} state but not fully representative of the local coordination environment and mobility properties of Cu^{II} ions under the oxidation step conditions, being associated to framework-coordinated Cu^{II} species. Based on the strong spectroscopic contrast between Cu^{I} and Cu^{II} moieties, the estimated Cu^{I}/Cu^{II} fractions obtained from this simple analysis are still reliable, as supported by the excellent LCF reproduction of the Cu(I) diagnostic peak at ca. 8983 eV. Yet, it is evident that more sophisticated data analysis approaches are required to pinpoint the details of Cu^{II} speciation under the oxidation step conditions.

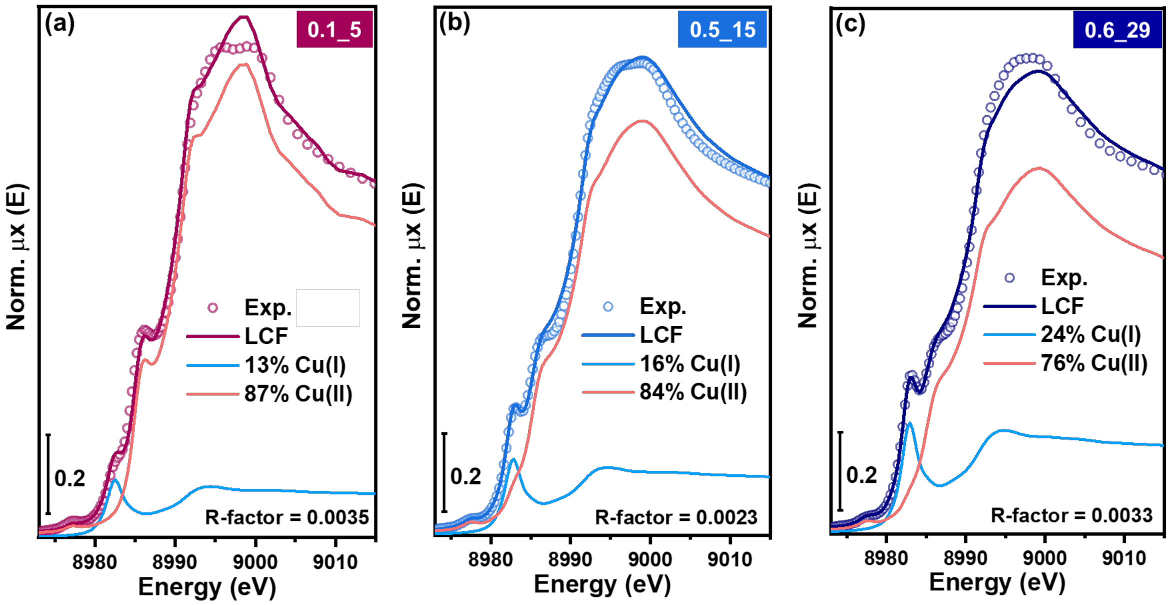

Figure S2: Comparison between experimental (colored circles) and LCF (thick colored lines) XANES spectra at the end of the oxidation step at 200 °C (40 min from exposure of the reduction state to 10% O_2/He gas flow, last point in Figure S1) for (a) 0.1_5, (b) 0.5_15, and (c) 0.6_29 Cu-CHA catalysts. In each panel, the scaled Cu^{I} and Cu^{II} components are also reported, as light blue and red thin lines, respectively, with indication of the corresponding Cu^{I}/Cu^{II} percentages and LCF R-factor values.

3 Wavelet Transform (WT) EXAFS analysis after the reduction and oxidation steps

3.1 *Methodological background*

The analysis of the Fourier Transform (FT)-EXAFS spectra is of undeniable utility to obtain accurate structural information about the local coordination environment of the absorber atom. However, if two or more groups of different atoms are localized at close distances around the absorber, their contributions in the direct R space overlap, becoming often indistinguishable.[10, 11] In this case, the classical analysis of the FT-EXAFS spectrum becomes rather uninformative. A possible solution to overcome this problem consists to combine the k^n weighted EXAFS signal and its FT representation localizing the scattering contribution of different atomic elements at different position in both the k and R spaces, respectively.[4, 10-14]

This task is nowadays possible and is performed by a modern spectral processing technique called Wavelet Transform (WT), which is able to provide a 2D representation of the EXAFS signal, revealing the signal features both in k and R-spaces simultaneously.

The WT of a general k^n-weighted EXAFS spectrum is defined as:

$$W^{\psi}(a,b) = \frac{1}{\sqrt{a}} \int_{-\infty}^{+\infty} dk'\, k'^{n} \chi(k') \psi^{*}\left(\frac{k'-b}{a}\right) \tag{S3.1}$$

This equation can be seen as the inner product between EXAFS $\chi(k)$ signal and a defined window function ψ, called mother wavelet or simply wavelet (where the apex ψ^* denotes the complex conjugate of ψ), which must decay at zero for higher values of $|k'|$. Here, the signal $\chi(k)$ is analyzed through a set of train-waves (wavelets) that are shifted by b units in the k-space and distorted by a factor a in order to take account of the local frequencies of the signal. The variables a and b are connected to k and R space by the following relations: $a = \eta/2R$ and $b = k$.

The mother function used in this work is the Morlet function, expressed in the following form:

$$\psi(k) = \frac{1}{\sqrt{2\pi\sigma}} exp\,(i\eta k) exp\,(-k^2/2s^2) \tag{S3.2}$$

Where i denotes the complex unit while η and s are two parameters regulating the wavelet resolution in R and k *s*paces, respectively. The selection of an appropriate set of these two parameters is critical and must be optimized in order to have the best visualization of the desired spectral features. In fact, it is worth

mentioning that the WT distributes the signal information over some *k-R* cells, usually named as uncertainty or Heisenberg boxes,[15] which are expressed for the Morlet wavelet as:

$$\left[k \pm \frac{\eta s}{\sqrt{2}R}\right] \times \left[R \pm \frac{R}{\sqrt{2}\eta s}\right] \quad \text{(S3.3)}$$

From eq. (S3.3), it is clear how the WT resolutions in k and R space result inversely proportional one to each other. Thus, having a good resolution in k space always involves a loss of information in the related R space, and *vice versa*.

Based on the encouraging results recently obtained in the study of Cu-zeolites[4, 12-14, 16] we applied a Wavelet Transform (WT) analysis on the EXAFS spectra collected at the end of the reduction and oxidation steps for each Cu-CHA sample, aiming at a complementary view on the EXAFS data assisting the quantitative analysis realized through the EXAFS fitting procedure. In particular, we exploited the fact that single scattering (SS) contributions stemming from different elemental neighbors are differently localized in the k-space, due to the behavior of their related backscattering amplitude factors F(*k*), shown in **Figure S3** for all the relevant atomic neighbors (O/N, Si/Al, Cu).

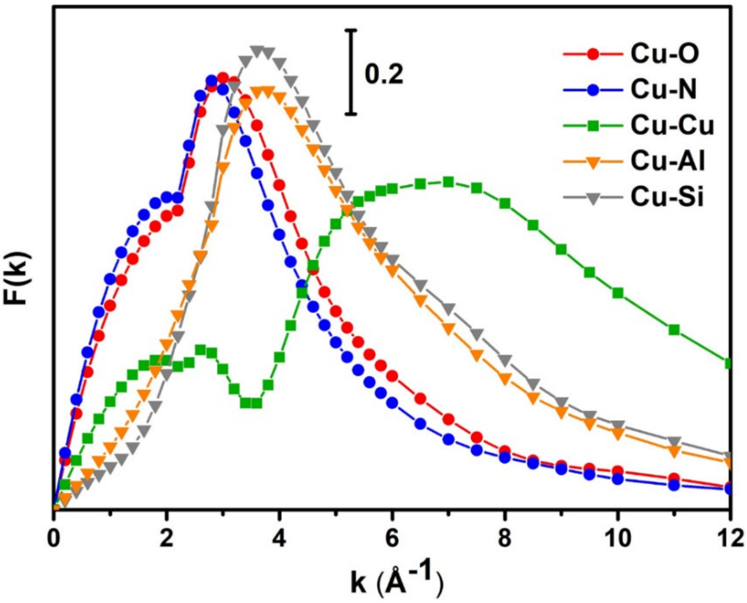

Figure S3: Backscattering amplitude function F(k) associated to the possible elements present in the investigated Cu-CHA catalysts under the experimental conditions at the reduction and oxidation steps, calculated using the FEFF code for Cu-Cu (green) Cu-O (red), Cu-N (blue), Cu-Al (orange) and Cu-Si (grey) Single Scattering (SS) paths. All the curves have been obtained by *ab initio* calculations using the FEFF6.01 software. In particular the curves referring to the O, N and Cu elements are retrieved from the scatterers belonging to the μ–η^2, η^2–peroxo diamino dicopper (II) $[Cu_2(NH_3)_4O_2]^{2+}$ complex, while the ones proper of the Al and Si curves are retrieved from the Cu-oxo model in ref.[14]

From **Figure S3** it is possible to note that the F(k) functions for SS paths involving O and N as well as Si and Al are completely overlapped (similar Z), making it impossible to discriminate O *vs* N or Al *vs* Si contributions using the WT representation. At the same time, also the O/N backscattering amplitudes are almost completely overlapped under the Al/Si ones. Therefore, O/N discrimination in presence of these elements also results rather complicated. Conversely, the Cu backscattering amplitude function is well separated from the precedent contributions making the Cu-Cu interaction possible to be visualized through the WT analysis, offering a powerful diagnostic tool to visually identify multimeric Cu species.

All the WTs reported in this work were performed selecting as k and R ranges within (0-12) $Å^{-1}$ and (0.5-4.5) Å, respectively. In the main text, we reported the magnified WTs in the 2–4 Å R-space range, where the signal interpretation based on the conventional FT-EXAFS approach is mostly complicated due to previously discussed overlapping contributions. The corresponding full-range WTs can be found in **Section 3.2**.

For all the computed WTs, we selected as wavelet parameter the values $\sigma = 1$ and $\eta = 7$. This choice aims at fulfilling the condition of *optimal resolution* at a given distance (Cu-Cu interatomic distances in the present case), as discussed by Funke *et al.* and Timoshenko *et al.*[10, 11] where it is showed that the best discrimination is reached for $\sigma = 1$ and $\eta = 2R_{opt}$, where R_{opt} is the real backscattering distance.

Moreover, to further assist the WT interpretation, we calculated the power density function $\Phi^R(\mathrm{k})$ for each analyzed sample/step (**Section 3.3**). $\Phi^R(k)$ is obtained by integrating the square modulus of the WT over the R-range 2-4 Å and it is defined by the following expression:

$$\Phi^R(\mathrm{k}) = \int_{R_{min}}^{R_{max}} dR' |W^{\Psi}(k, R')|^2 \qquad \text{(S3.4)}$$

Where R_{min} =2 Å, R_{max} = 4 Å, and $W^{\Psi}(k, T)$ is the wavelet transform representation of the EXAFS signal depending on the mother function used, corresponding here to the Morlet function, eq. (S3.2). The resulting $\Phi^R(k)$ function is a k-only-dependent function, the maxima of which determine the signals localization in the k space and can be directly compared with the F(k) functions, while facilitating the comparison between different samples.

3.2 *Full-range WT-EXAFS maps after reduction and oxidation steps*

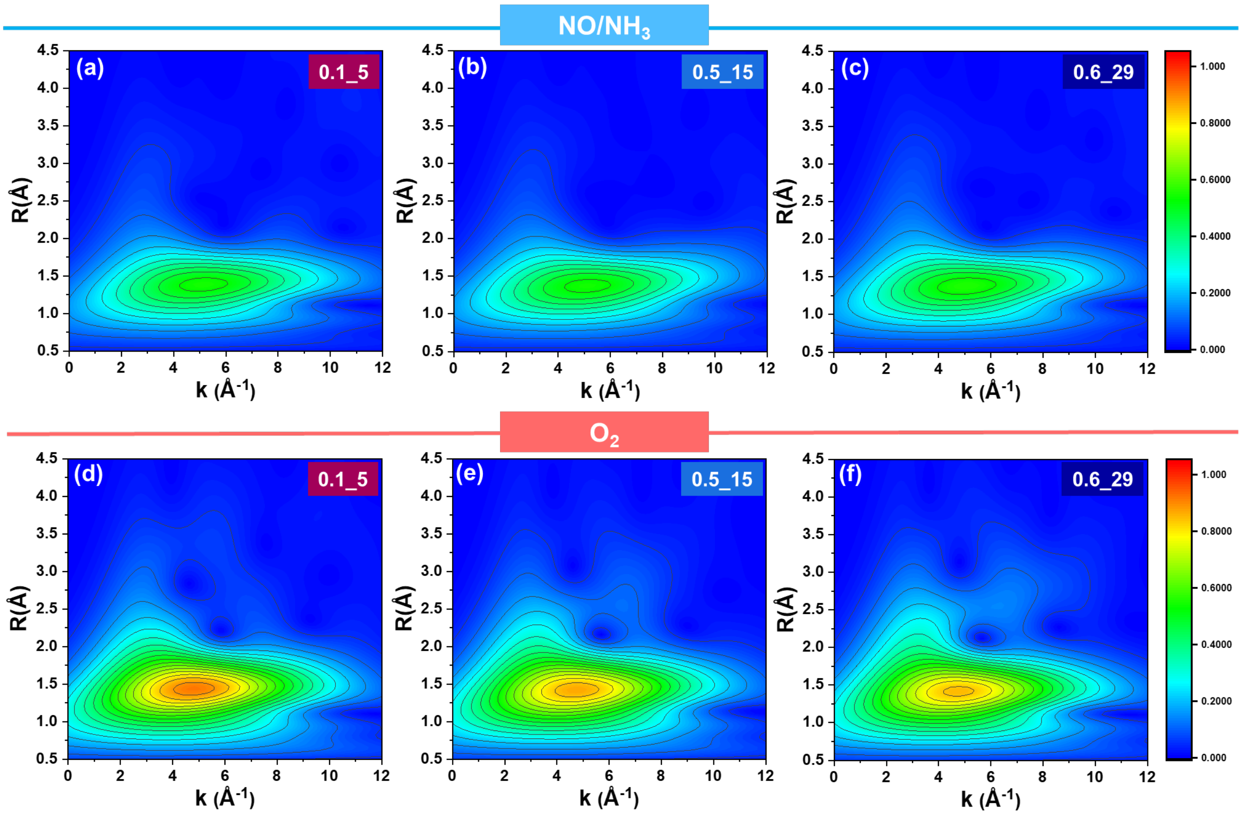

Figure S4: Moduli of the full-range EXAFS-WT maps for the three investigated Cu-CHA catalysts, (a-c) at the end of the reduction step and (d-e) at the end of the oxidation step.

3.3 *Density power functions after reduction and oxidation steps*

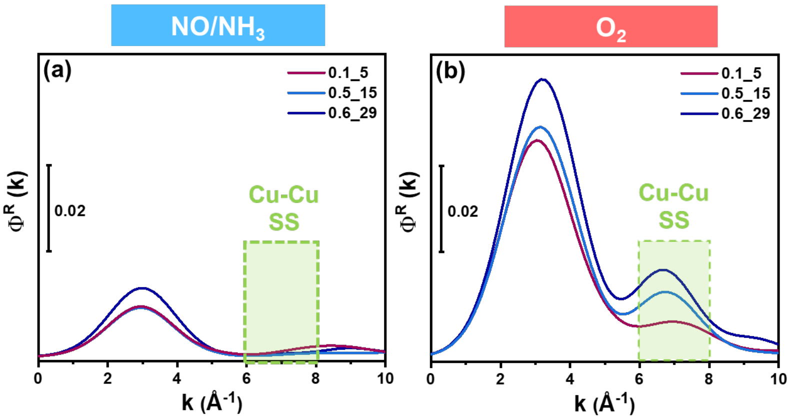

Figure S5: Density power functions Φ^R calculated in the 2.0-4.0 Å R-space range for the WT EXAFS representations the three investigated Cu-CHA catalysts (a) at the end of the reduction step and (b) at the end of the oxidation step. k-space range diagnostic for Cu-Cu SS contributions, based on backscattering amplitude factors $F(k)$ reported in Figure S3, is highlighted by a dashed green box.

4 Conventional EXAFS fitting results

4.1 Methods and adopted models for conventional EXAFS fitting

Conventional R-space EXAFS fits for the reduction state of all the investigated catalysts were performed using the $[Cu^{I}(NH_3)_2]^+$ complex input geometry (**Table S2**, top), based on the observed XANES features. For preliminary EXAFS fitting after the oxidation step of the two catalysts displaying evident Cu-Cu scattering contribution in EXAFS WT maps, namely 0.5_15 and 0.6_29, we relied on the previously reported DFT-optimized geometry of side-on μ-η^2, η^2-peroxo diamino dicopper (II) $[Cu_2(NH_3)_4O_2]^{2+}$ complexes (**Table S3**, top).[4]

Phases and backscattering amplitudes have been computed by FEFF6.01,[17] as implemented within the Artemis software from the Demeter package.[8] The adopted fitting models included all the single (SS) and the most intense multiple (MS) scattering paths contributing in the R-space fitting range, 1.0-4.0 Å. The sole exception was represented by the SS and MS contributions involving H atoms, neglected because of their low scattering amplitude.

In order to limit the number of optimized variables, all the SS and MS paths were refined using the same passive amplitude reduction factor (S_0^2) and the same energy shift parameter (ΔE). The coordination numbers were set according to the employed input structural models. In the fitting model, all the SS paths were identified, and the related interatomic distances from the Cu absorber were parametrized using radial shifts ΔR_x (with x = O, N and Cu) guessed starting from an initial value of 0 Å (i.e., no structural variation from the initial geometry). In parallel, each SS was parameterized with independent Debye-Waller (DW) factors, σ^2_x (with x = O, N, Cu), initialized on the basis of previously reported fitting results. MS contributions were defined as a function of ΔR_x and σ^2_x optimized for the relevant SS paths, with radial shift parameters derived from geometrical considerations and DW factors computed as $\sigma^2_{MS} = [\Sigma_i (\sigma^2_{x,i})^2]^{1/2}$.

4.2 *Conventional EXAFS fitting results after the reduction step*

Table S2: Results of the fits executed on the k^2-weighted FT-EXAFS spectrum (transformed in the 2.4-12.0 Å^{-1} k-range) of the three Cu-CHA catalysts at the end of the reduction step (NO/NH_3 at 200 °C). The model of the $[Cu^I(NH_3)_2]^+$ complex used as initial guesses for the fitting procedure is also illustrated, with the corresponding initial interatomic distances reported in parentheses in the Table. Color code in the structures: Cu, green; H, white; N, blue. Parameter values set in the fit are reported as underlined. In particular, the S_0^2 parameter was initially guessed for 0.5_15, and subsequently set in the fits for 0.1_5 and 0.6_29, to guarantee a reliable comparison among the fitting results over the three Cu-CHA catalysts.

Conventional EXAFS fitting results – reduction step – $[Cu^I(NH_3)_2]^+$ model

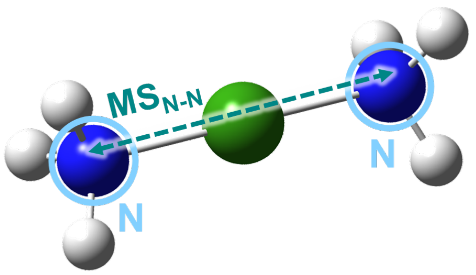

EXAFS parameters	Best-fit values 01_5	Best-fit values 05_15	Best-fit values 06_29
$N°_{par}/N°_{ind}$	3/18	4/18	3/18
R-factor	0.015	0.014	0.016
S_0^2	0.96	0.96 ± 0.06	0.96
ΔE (eV)	-2.1 ± 0.7	-2.5 ± 0.6	-2.3 ± 0.7
$<R_N>$ (Å)	1.912 ± 0.005 (1.912)	1.912 ± 0.004 (1.912)	1.917 ± 0.005 (1.912)
σ^2_N (Å^2)	0.0047 ± 0.0005	0.0045 ± 0.0003	0.0043 ± 0.0004

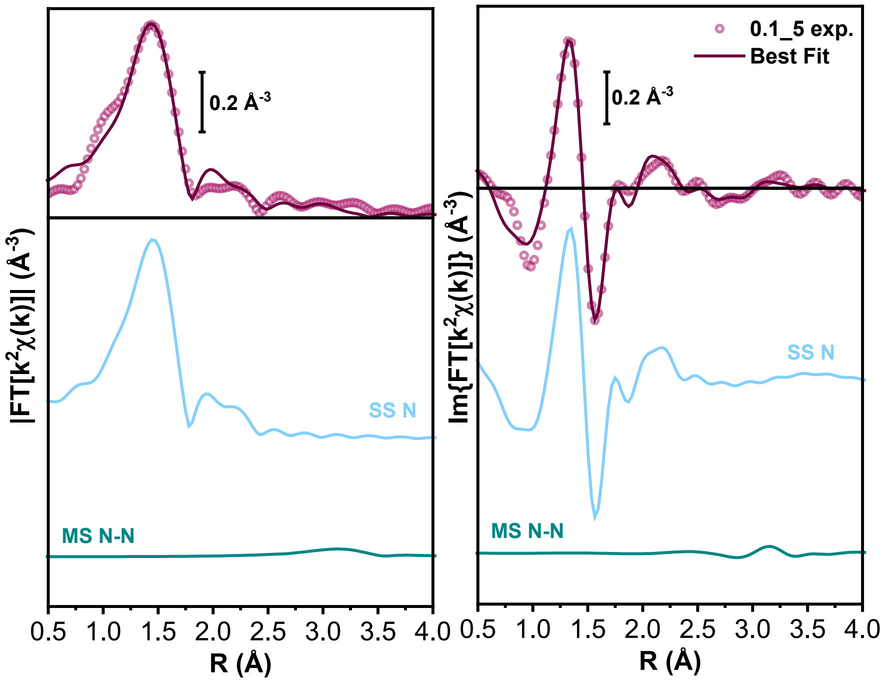

Figure S6: EXAFS fit results for the 0.1_5 Cu-CHA sample at the end of the reduction step. The experimental and best-fit FT-EXAFS spectra are reported in both modulus and imaginary part, in left and right panel, respectively, together with the individual SS and MS contributions to the total signal, vertically shifted for clarify. Color code of the scattering paths: SS N, light blue; MS N-N, cyan.

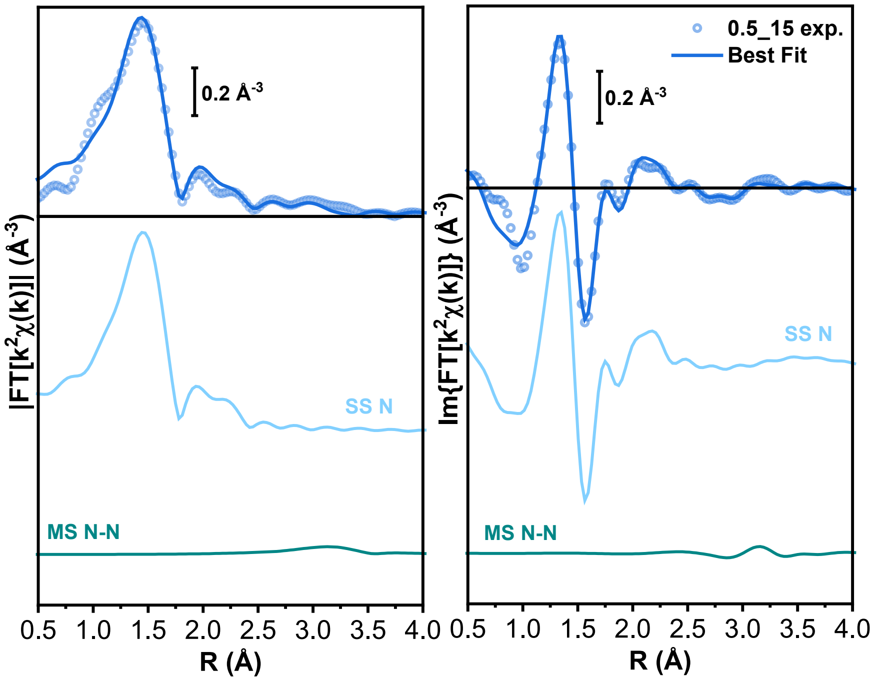

Figure S7: EXAFS fit results for the 0.5_15 Cu-CHA sample at the end of the reduction step. The experimental and best-fit FT-EXAFS spectra are reported in both modulus and imaginary part, in left and right panel, respectively, together with the individual SS and MS contributions to the total signal, vertically shifted for clarify. Color code of the scattering paths: SS N, light blue; MS N-N, cyan.

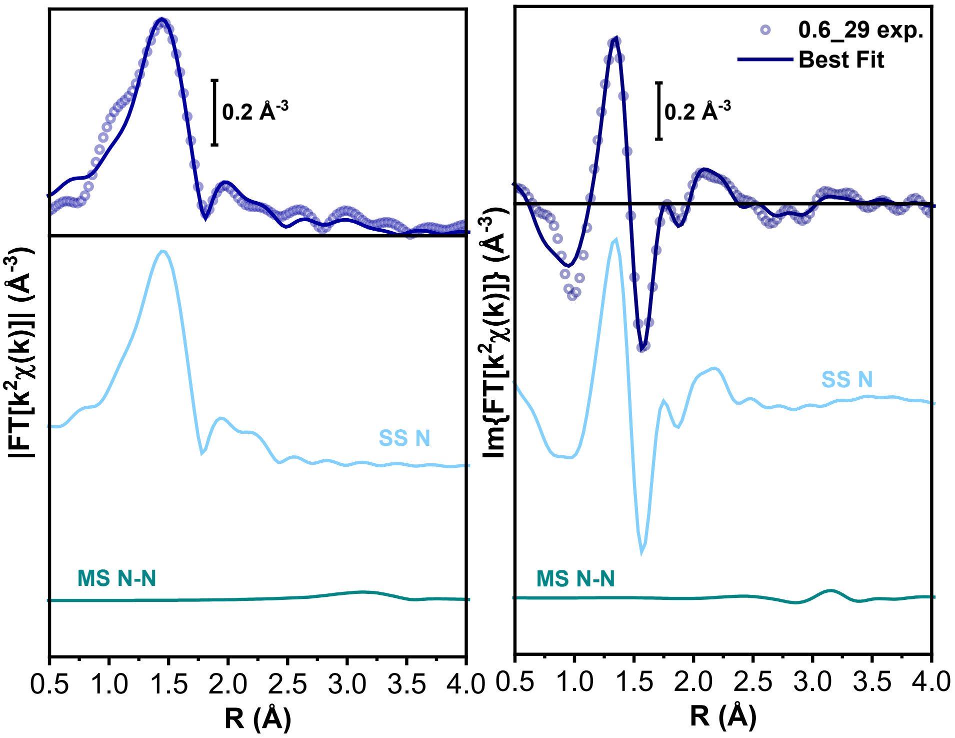

Figure S8: EXAFS fit results for the 0.6_29 Cu-CHA sample at the end of the reduction step. The experimental and best-fit FT-EXAFS spectra are reported in both modulus and imaginary part, in left and right panel, respectively, together with the individual SS and MS contributions to the total signal, vertically shifted for clarify. Color code of the scattering paths: SS N, light blue; MS N-N, cyan.

4.3 *Preliminary results from conventional EXAFS fitting after the oxidation step for 0.5_15 and 0.6_29*

Table S3: Results of the fits executed on the k^2-weighted FT-EXAFS spectrum (transformed in the 2.4-12.0 Å^{-1} k-range) of the 0.5_15 and 0.6_29 Cu-CHA catalysts at the end of the oxidation step (10% O_2/He at 200 °C). The model of the side-on μ–η^2, η^2–peroxo diamino dicopper (II) $[Cu_2(NH_3)_4O_2]^{2+}$ complex used as initial guesses for the fitting procedure is also illustrated, with the corresponding initial interatomic distances reported in parentheses in the Table. Color code in the structures: Cu, green; H, white; N, blue O, red. Parameter values set in the fit are reported as underlined. In particular, the σ^2_N parameters were set to the best-fit values obtained for the corresponding catalysts from EXAFS analysis at the end of the reduction step (see Table S2).

Conventional EXAFS fitting results – oxidation step 0.5_15, 0.6_29
side-on μ–η^2, η^2-peroxo diamino dicopper (II) $[Cu_2(NH_3)_4O_2]^{2+}$ model

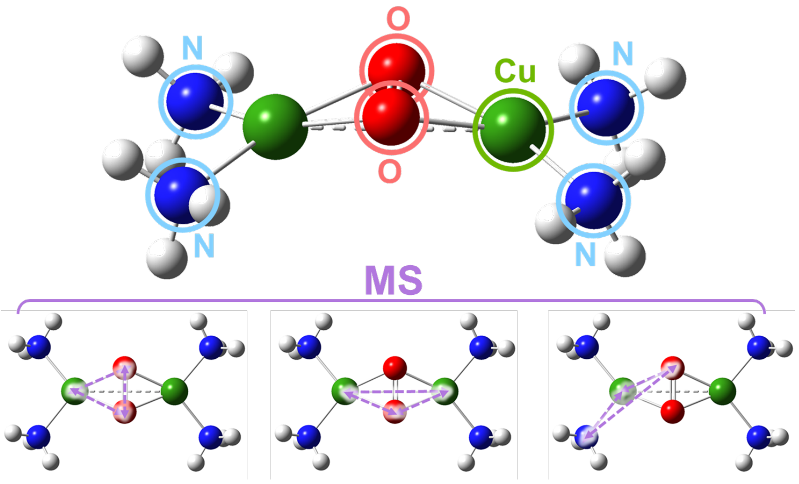

EXAFS parameters	Best-fit values 05_15	Best-fit values 06_29
N°$_{par}$/N°$_{ind}$	7/18	7/18
R_{factor}	0.019	0.03
S_0^2	0.84 ± 0.06	0.84 ± 0.08
ΔE (eV)	1 ± 1	2 ± 2
<R_O> (Å)	1.908 ± 0.009 (1.955)	1.902 ± 0.012 (1.955)
<R_N> (Å)	2.06 ± 0.02 (2.03)	2.05 ± 0.03 (2.03)
R_{Cu} (Å)	3.40 ± 0.05 (3.40)	3.40 ± 0.05 (3.40)
σ^2_O (Å^2)	0.003 ± 0.001	0.003 ± 0.002
σ^2_N (Å^2)	<u>0.0045</u>	<u>0.0043</u>
σ^2_{Cu} (Å^2)	0.014 ± 0.007	0.011 ± 0.006

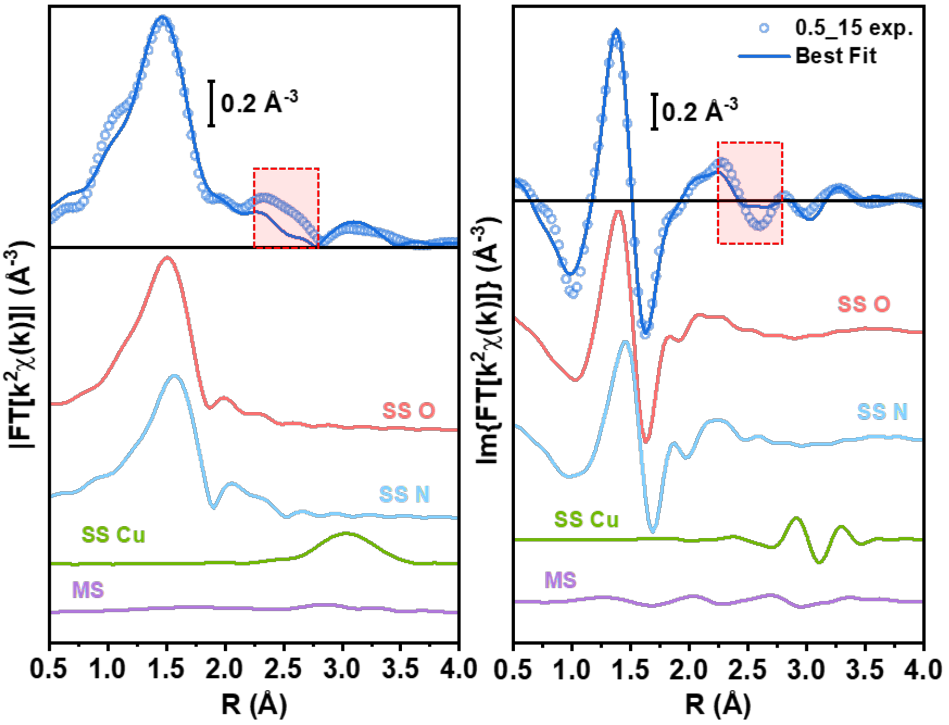

Figure S9: Preliminary EXAFS fit results for sample 0.5_15 at the end of the oxidation step. The experimental and best-fit FT-EXAFS spectra are reported in both modulus and imaginary part, in left and right panels, respectively, together with the individual SS and MS contributions to the total signal. Color code of the scattering paths: SS O red; SS N, light blue; SS Cu, green; MS, violet. The red dashed boxed highlight the local lack of fit observed in the 2.25-2.75 Å (phase-uncorrected) range, with cannot be accounted by the presence of the minor contribution from $[Cu^{I}(NH_3)_2]^+$ complexes.

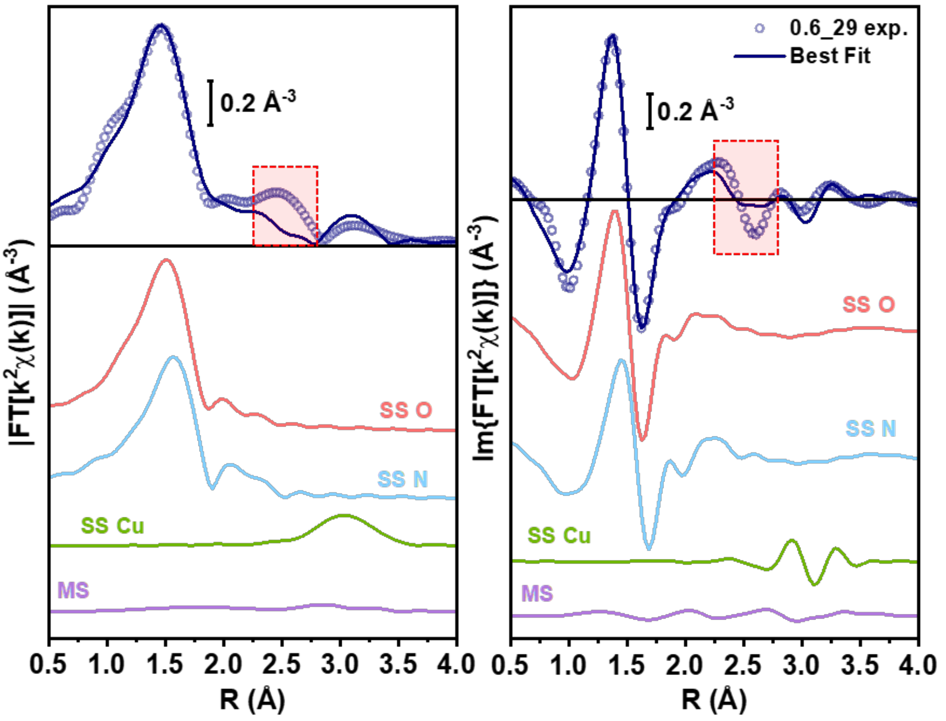

Figure S10: Preliminary EXAFS fit results for sample 0.6_29 at the end of the oxidation step. The experimental and best-fit FT-EXAFS spectra are reported in both modulus and imaginary part, in left and right panels, respectively, together with the individual SS and MS contributions to the total signal. Color code of the scattering paths: SS O red; SS N, light blue; SS Cu, green; MS, violet. The red dashed boxed highlight the local lack of fit observed in the 2.25-2.75 Å (phase-uncorrected) range, with cannot be accounted by the presence of the minor contribution from $[Cu^{I}(NH_3)_2]^+$ complexes.

5 EXAFS fitting after the oxidation step through a FEFF-based Machine Learning Indirect Approach

5.1 Methodological background

The FEFF-based fitting procedure provided by the Artemis code (i.e., IFEFFIT) is a well establish methodology employed to extract quantitative structural information from an experimental EXAFS spectrum. During the years, this approach allowed to achieve several important results, especially in the field of catalysis, certifying its validity and imposing as one of the main codes for the EXAFS analysis. However, some drawbacks in this methodology can be identified. First, the Artemis fitting procedure is based on the *ab initio* evaluation of the effective backscattering path amplitudes, phases and electron mean free paths of the EXAFS signals (SS and MS) extracted from a user-defined input structure. It follows that these functions do not show any dependence from the molecular geometry but only on k. This fact has a strong repercussion on the optimization procedure enabling the refinement of only small variations of the interatomic distances and limiting the possibility to obtain an accurate estimation of the molecular bond angles, which are usually neglected in the *classical* (FEFF-based) Artemis analysis. A second issue regards the parametrization of the effective scattering length of the MS paths involved in the EXAFS fit. Typically, these quantities are expressed as a function of the ones of the major SS processes affecting the fit. This choice clearly reduces the number of parameters involved and limits the fitting correlations but it can determine also a drastic simplification of the model or could cause the arising of complex long expressions making the fitting procedure sometimes difficult to handle. Recently, all the stated problems have been overcame by the introduction of a novel fitting procedure, described in ref.[18] The latter foresees the approximation of each theoretical $\chi_i(k)$ path signals (SS and MS) through the generation of a corresponding Machine-Learning-based interpolation function, under the variation of user-defined sets of n-structural parameters: $\boldsymbol{p} = (p_1, \ldots, p_n)$. The perturbation of each element of $\boldsymbol{p}$ (e.g., bond distances, angles of the input structure, etc.) is then reflected automatically in the changes of the effective backscattering amplitude, phases and electron mean free path. These terms, in turn, determine a modification of the correspondent path signal χ_i allowing to have the precise description for every perturbed geometry. In order to clarify this concept, **Figure S11** illustrates the variation of the single EXAFS signal for two representative SS and MS paths, deriving from the *μ-η²,η²*-peroxo diamino dicopper(II) model $[Cu_2(NH_3)_4O_2]^{2+}$ compound. Herein three representative values of the angular parameter Δα of 0°, 15° and 30° have been selected. For more information concerning the molecular parameterization and the atoms indexing, the reader is referred to **Figure S12**.

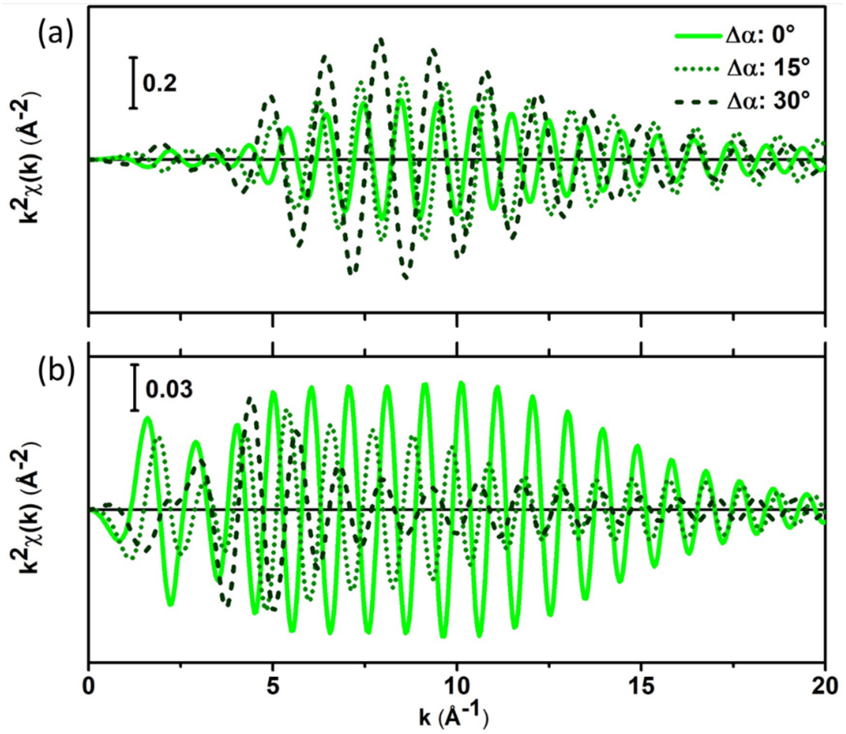

Figure S11: Plot of two representative SS and MS (3 legs) EXAFS signals, namely (a) $Cu_1 \rightarrow Cu_6$ and (b) $Cu_1 \rightarrow Cu_6 \rightarrow O_5 \rightarrow Cu_6$ as a function of the variation of the Δα parameter affecting the dihedral α angle of the μ-η^2,η^2-peroxo diamino dicopper(II) model structure. For more information about the atoms indexing and parameterization, the reader can refer to Figure S12.

The generation of a set ML-approximated χ_i functions is a multi-step process, which for sake of clarity will be discussed in the following paragraph.

The first step consists into identifying a set of structural parameters which needs to be further refined through the fitting routine. Then, a first multidimensional coarse grid, constituted by all possible parameters combinations involving the maxima and minima values plus the null deformations (i.e., $\boldsymbol{p} = (p_1 = 0, \ldots, p_n = 0)$), is generated. For each grid-point, a FEFF6.01 input file is created and run. Afterwards, for each molecular configuration, the rank intensities (i.e., the curve wave amplitudes) associated to each path are analyzed in order to select all the not equivalent paths having a strong impact on the fitting routine independently from the geometry. Once this step is completed, a finer grid of deformations is generated recurring to the Improved Latin Hypercube method of sampling (IHS).[18, 19] For each new structure, the FEFF code is executed and only the FEFF files related to those paths identified through the coarse grid analysis are considered. As a result, each significant path will be characterized by a proper dataset containing its related signals evaluated for every deformation of the IHS grid. Each of them is then approximated for a continuous variation of the selected set of deformations through a ML-regressor algorithm.

Being in possess of the most significant χ_i path functions, it is possible to weight each of them for their related DW exponential term, S_0^2 factors and, if necessary, to apply a shift in energy. Finally, the resulted path functions are summed together in order to generate the total theoretical EXAFS signal. The refinement is instead realized by minimizing the R-factor between the total theoretical and experimental spectrum while, similarly to the EXCURVE code, the uncertainties associated to the optimized parameters are evaluated using the Fisher test analysis fixing a significate level to 68%.[18, 20]

5.2 *Fitting model and parametrization strategy*

In the main text and in Section 4.3, we indicated that a probable improvement of the EXAFS spectra, acquired during the oxidation step, can be achieved accounting for a minor presence of $[Cu^I(NH_3)_2]^+$ species as well as for a bimodal distribution of the Cu-Cu interatomic distances of the *μ-η²,η²*-peroxo diamino dicopper(II) complexes between 3.0 and 3.4 Å. Based on these considerations, hereafter we will discuss in detail, the procedure and the results of the EXAFS fit spectra concerning the 0.5_15, 0.6_29 and 0.1_5 samples acquired during the oxidation step.

The fitting procedure employed in the analysis of these data has been realized through an *in house* Python code exploiting the PyFitIt libraries.[19] The resulting model is a hybrid between the *classic* one reported in **Section 4.1** and the new methodology described in in **Section 5.1**. In particular, the total theoretical EXAFS signal has been generated as the sum of three EXAFS contributions as follow:

$$\chi^{THEORY} = w_1\chi_1 + w_2\chi_2 + w_3\chi_3 \quad \text{S3.5}$$

Where χ_1 and χ_2 are the total theoretical EXAFS signals referring to the DFT optimized $[Cu^I(NH_3)_2]^+$ (model ❶) and *μ-η²,η²*-peroxo diamino dicopper(II) $([Cu_2(NH_3)_4O_2]^{2+})^+$ (model ❷) structures, obtained through the *classical* approach. The parameterization of these two structures followed the ones previously described in **Section 4.1**. On the other hand, signal χ_3 has been obtained through the ML-based methodology. The latter appears necessary to treat precisely the variations of the effective backscattering amplitudes, phases and electron mean free path of the main SS and MS signals characterizing the total EXAFS spectrum of the *μ-η²,η²*-peroxo diamino $[Cu_2(NH_3)_4O_2]^{2+}$ complex, under the variation of the Cu-N/O distances and of the angle α (model ❸), see **Figure S12**. The coefficients w_1, w_2 and w_3 represent

the fraction of each compound (i.e., $[Cu^{I}(NH_3)_2]^+$, quasi-planar *μ-η²,η²*-peroxo diamino dicopper(II) $[Cu_2(NH_3)_4O_2]^{2+}$ and its bent version) obeying to the *mass balance condition* constraint: $w_1 + w_2 + w_3 = 1$.

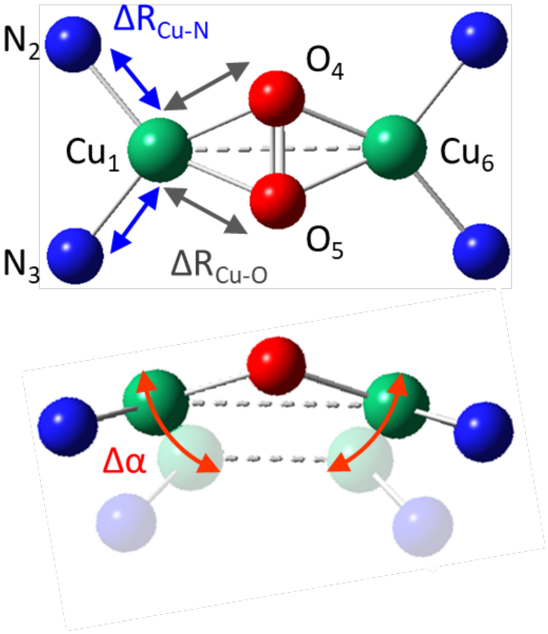

Figure S12: Representation of the μ-η²,η²-peroxo diamino dicopper(II) $[Cu_2(NH_3)_4O_2]^{2+}$ complex geometry, model ❸ together with the set of structural parameters (deformations) selected to fit the experimental EXAFS spectra showed in Figure 4 of the main text. For sake of clarity the H atoms have been removed from the representation since their contribution to the total EXAFS signal is negligible. Color code: O: red, N: blue, Cu: green.

The generation of the χ_3 signal was based on the following procedure.

Starting from the DFT optimized $[Cu_2(NH_3)_4O_2]^{2+}$ structure, three parameters have been selected for the refinement (see **Figure S12**): the four Cu_1–N and Cu_1–O bonds lengths: $\Delta R_{Cu\text{-}N}$ and $\Delta R_{Cu\text{-}O}$ and finally the variation of the $\Delta\alpha$ angle influencing the relative Cu_1–Cu_6 distance. The *coarse* grid associated to these parameters was generated assuming a range of variation for $\Delta R_{Cu\text{-}N}$ and $\Delta R_{Cu\text{-}O}$ within −0.12 and +0.12 Å, while the Δα angular parameter was perturbed within 0° and +30° respect to the initial structure, causing a maximum variation of the dihedral α angle of 60° (i.e. 2Δα for Δα=30°).

To select the most relevant paths required for the fitting procedure, we fixed the rank cutoff at 15% for the curve-wave amplitude ratios associated to each generated path. Under this constraint, the list of the most intense not *equivalent* paths was composed by the following 5 items: Cu→O→Cu (SS signals including the Cu_1→O_4 and Cu_1→O_5 paths), Cu→N→ Cu (SS signals including the Cu_1→N_2→Cu_1 and Cu_1→ N_3→ Cu_1 paths), Cu→Cu→ Cu (SS signal involving the Cu_1→Cu_6 →Cu_1 path), Cu→O→N→Cu (3 legs MS signals including the Cu_1→O_4→N_3→Cu_1 and Cu_1→O_5→N_2→Cu_1 paths and their reversal configurations due to the

inverted time symmetry[18]), Cu→O→O→Cu (3 legs MS including the $Cu_1 \rightarrow O_3 \rightarrow O_4 \rightarrow Cu_1$ and $Cu_1 \rightarrow O_4 \rightarrow O_3 \rightarrow Cu_1$ paths), and finally Cu→Cu→O→Cu (3 legs MS including the $Cu_1 \rightarrow Cu_6 \rightarrow O_4 \rightarrow Cu_1$ and $Cu_1 \rightarrow Cu_6 \rightarrow O_4 \rightarrow Cu_1$ paths together with their time-reversal versions). Herein the ML interpolation approach, represented by the Extra Trees regressor[19] was exploited to associate to each group of items the related, unique, path signal, approximated for the changes involving the $\Delta R_{Cu\text{-}N}$, $\Delta R_{Cu\text{-}O}$ and $\Delta\alpha$ parameters. In particular, the latter was realized by sampling the parameters space ($\Delta R_{Cu\text{-}N}$, $\Delta R_{Cu\text{-}O}$, $\Delta\alpha$) with 500 points, selected by means of the IHS scheme.

In the proposed analysis, we assumed that model ❸ was characterized by the same variations in interatomic distances from Cu to O and N atoms as in model ❷, as well as by the same DW values: σ_N^2, σ_O^2 and σ_{Cu}^2. Under these conditions, the total number of fitting parameters resulted be equal to 10, assuming a common, fixed S_0^2 parameter of 0.9. Although high, the number of fitting parameters is below the limit imposed by the maximum number of independent points ($2\Delta k\Delta R/\pi \approx 18$) and was used as a first screening regarding the possibility to monitor the reliability of the fit based solutions. In particular, we observed that the DW terms assumed the values of 0.004 Å^2 for the N and O atoms while for the Cu-Cu interaction this quantity is refined to 0.007 Å^2, respectively. Both of these results are in accordance with previous findings for these kind of systems[2, 4, 21, 22] and, within their related uncertainty ranges, with the results showed in **Table S3**, although with a lower value. We attribute these discrepancies to the lack of fit evident in the previous attempt. Since a fit with 10 parameters would cause the increase of the correlation among the fitting variables and the consequent enlargement of their uncertainty range, we decided to maintain fixed DW parameters to these last (feasible) fitted values, reducing the fitting variable to 7 over a maximum of 18 and consequently the parameters correlations.

5.3 Detailed report on fitting results

Table S5 reports the EXAFS fit results obtained at the end of the oxidation step for the three samples. The experimental FT-EXAFS spectra of the high Si/Al samples are perfectly reproduced by the best-fit curve in the whole R region. For both samples the lack of fit previously found in the reproduction of the 2.0-2.8 Å feature is now fixed by the signal due to model ❸ while the reproduction of the one peaking at ca. 3.4

Å is perfectly replicated by the signal due to model ❷. Moreover, in both samples, the R_{Cu3} value reported in **Table S5**, related to the interatomic Cu-Cu distance of the μ-η^2,η^2-peroxo diamino dicopper(II), model ❸, is similar and falls at ca. 3.0 Å. This means that the reproduction of the experimental EXAFS spectra of both samples is performed by the multicomponent EXAFS fit using the same three structural models. However, a significant difference between the two fits lies in the values of the refined components weights. Specifically, the EXAFS fitting results for the 0.6_29 sample point to a higher amount of model ❷ species over model ❸ ($w_2 = 0.31$; $w_3 = 0.43$). Conversely, in the case of the 0.5_15 sample, the EXAFS-determined weights for the two μ-η^2,η^2-peroxo diamino dicopper(II) species seems to be almost equivalent ($w_2 = 0.41$; $w_3 = 0.38$). Lastly, it is interesting to note that the w_1 values, that provides an estimation of the weight component related to residual $[Cu^I(NH_3)_2]^+$ species (model ❶), follow the same trend observed employing the XANES LCF analysis, as discussed in **Section 2.** Indeed, the presented EXAFS analysis confirms that Cu^I fraction is the lowest in 0.1_5 and the highest in 0.6_29, while it assumes an intermediate value for the 0.5_15 sample.

The experimental FT-EXAFS spectra of the 0.1_5 sample is satisfactorily reproduced, although the fitting quality is slightly lower than in the other two cases, especially in the region above 3 Å in both magnitude (Figure 4 of the main text) and imaginary part plots (**Figure S13**). This result is not surprising since the WT analysis shows that, for this catalyst, the Cu-Cu scattering contribution is weaker and has a different morphology than in the case of the high Si/Al samples. Moreover, as previously discussed in **Section 2**, this low Si/Al sample seems to deviate from the model proposed by Paolucci *et al.*,[9] pointing to the Cu-density as the main descriptor to predict Cu-speciation and Cu^I/Cu^{II} balance under the oxidation step conditions.

Interestingly, by looking at the $\Delta\alpha_3$ value reported in **Table S5** (2.8°) for the 0.1_5 sample, it is clear that the presence of a second μ-η^2,η^2-peroxo diamino dicopper(II) species with shorter Cu-Cu interatomic distance is not needed to improve the EXAFS fit in this case. The fitting model is ultimately adjusted to use only a single type of dicopper(II) moiety, with an average Cu-Cu separation of $R_{Cu\_3} \sim R_{Cu\_2} \sim 3.4$ Å. In parallel, the w_1 value found by the EXAFS fit analysis for this sample is still in agreement with the LCF coefficients reported in **Section 2**, being the lowest among the Cu-CHA samples.

These results point towards the presence of long-range Cu-Cu scattering interactions also for 0.1_5 case, while for this sample the 'bent' μ-η^2,η^2-peroxo diamino dicopper(II) with Cu-Cu distance of ca. 3.0 Å is found to be largely absent. Yet, considering the slightly lower fit quality (but still indicating a good matching with the experimental curve), and the fact that the small distance difference between R_{Cu2} = 3.39 Å and R_{Cu3} = 3.38 Å leads to a slight dampening of EXAFS features due to antiphase effects, it is still plausible that a Cu^{II} species with similar structure as the model ❷ one, but with different chemical nature, could form for the 0.1_5 sample. Plausible hypotheses to be investigated in future studies include two proximal Cu(II) monomeric species or other dicopper(II) dimers, with a four-fold coordination of the Cu(II) ions and a Cu-Cu separations of ca. 3.4 Å, not yet explored computationally.

Table S4: Results of the fits executed on the k^2-weighted FT-EXAFS spectrum (transformed in the 2.4-12.0 Å^{-1} k-range) of Cu-CHA after the oxidation step in O_2 at 200 °C. The structures of ❶ ($[Cu^I(NH_3)_2]^+$), ❷ and ❸ ('planar' and 'bent' $[Cu_2(NH_3)_4O_2]^{2+}$) model components, used as initial guesses for the fitting procedure, are also illustrated. Color code in the structures: Cu, green; H, white; N, blue. Colored circles highlight the different shells of neighboring atoms included in the fitting model. Parameter values set in the fit are reported as underlined.

Results of ML-assisted EXAFS fits for the oxidation step

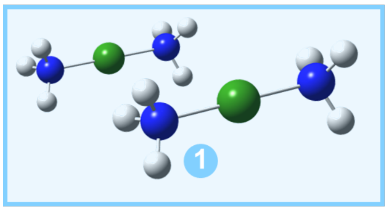

$[Cu(NH_3)_2]^+$

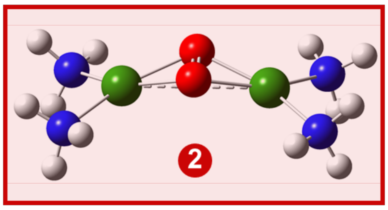

$[Cu_2(O_2)(NH_3)_4]^{2+}$ ***'planar'***

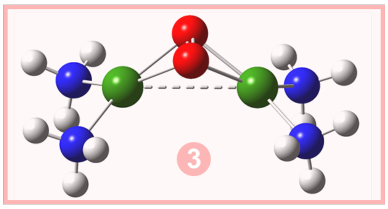

$[Cu_2(O_2)(NH_3)_4]^{2+}$ ***'bent'***

Fit parameters	Best-fit values 01_5	Confidence Intervals (68%)	Best-fit values 05_15	Confidence Intervals (68%)	Best-fit values 06_29	Confidence Intervals (68%)
$N°_{par}/N°_{ind}$	7/18	/	7/18	/	7/18	/
R-factor	0.007	/	0.005	/	0.008	/
S_0^2	<u>0.9</u>	/	<u>0.9</u>	/	<u>0.9</u>	/
ΔE (eV)	-3.49	-5.46 ÷ -1.38	-2.86	-4.62 ÷ -1.31	-2.61	-4.95 ÷ 0.61
R_{N1} (Å)	<u>1.91</u>	/	<u>1.91</u>	/	<u>1.91</u>	/
$<R_{O2,3}>$ (Å)	1.922 (1.955)	1.91 ÷ 1.95	1.914 (1.955)	1.90 ÷ 1.94	1.911 (1.955)	1.89 ÷ 1.94
$<R_{N2,3}>$ (Å)	2.028 (2.03)	2.00 ÷ 2.06	2.027 (2.03)	2.01 ÷ 2.05	2.029 (2.03)	2.00 ÷ 2.06
R_{Cu2} (Å)	3.39 (3.44)	3.32 ÷ 3.51	3.36 (3.44)	3.32 ÷ 3.43	3.34 (3.44)	3.32 ÷ 3.35
$\Delta\alpha_3$ (°)	2.8[b]	0 ÷ 10.3	15.5	12.6 ÷ 19.2	13.7	10.9 ÷ 16.8
R_{Cu3} (Å)[a]	3.38 (/)[b]	3.19 ÷ 3.44	3.06 (/)	2.97 ÷ 3.12	3.03 (/)	2.99 ÷ 3.17
σ^2_O (Å^2)	<u>0.004</u>	/	<u>0.004</u>	/	<u>0.004</u>	/
σ^2_N (Å^2)	<u>0.004</u>	/	<u>0.004</u>	/	<u>0.004</u>	/
σ^2_{Cu} (Å^2)	<u>0.007</u>	/	<u>0.007</u>	/	<u>0.007</u>	/
w_1	0.18	0.12 ÷ 1.04	0.21	0.22 ÷ 0.89	0.26	0.19 ÷ 0.93
w_2	0.43	0.11 ÷ 0.66	0.41	0.19 ÷ 0.57	0.31	0.19 ÷ 0.68
w_3	0.39	0.04 ÷ 0.81	0.38	0.12 ÷ 0.69	0.43	0 ÷ 0.64

[a] R_{Cu3} is calculated from the optimized value of $\Delta\alpha_3$ based on geometrical considerations.

[b] For 0.1_5, the optimized value of $\Delta\alpha_3$ and the corresponding R_{Cu3} distance correspond with the characteristic values for model ❷ within the technique accuracy, pointing to the presence of the sole planar $[Cu_2(O_2)(NH_3)_4]^{2+}$ complex in this catalyst. For this reason, the corresponding component for 0.1_5 is referred to as ❷' in Figure 4 (main text) and **Figure S13**.

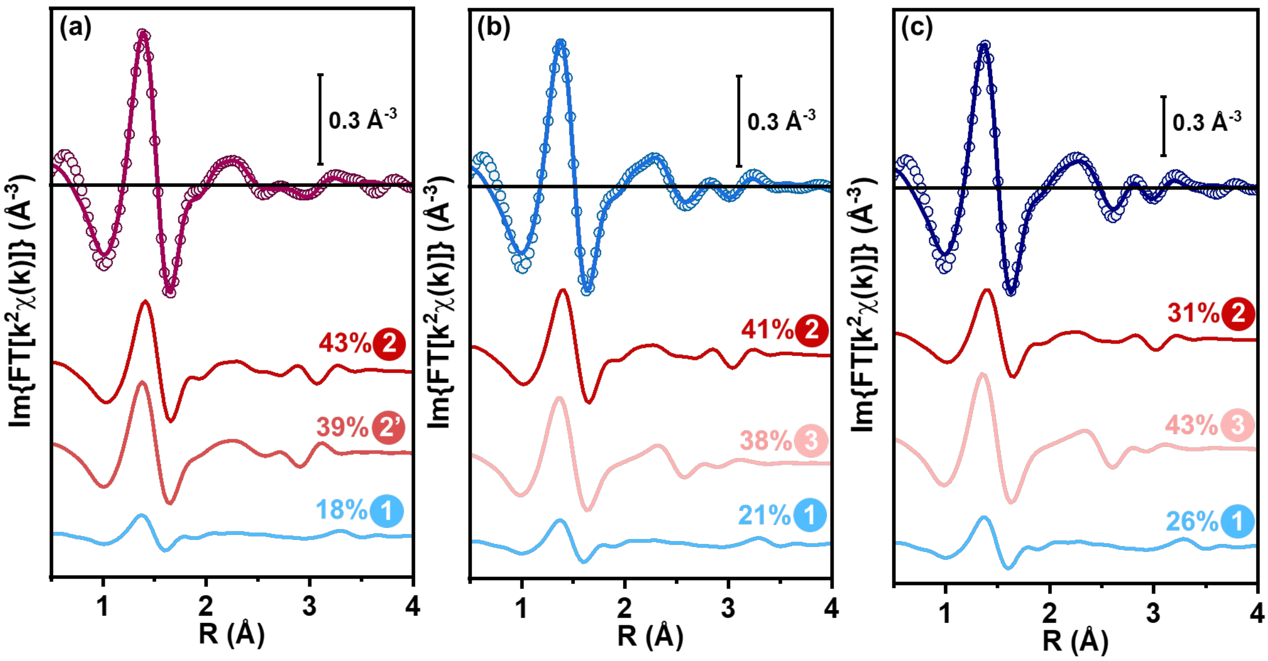

Figure S13: Comparison between imaginary parts of experimental and best-fit FT-EXAFS spectra for (a) 0.1_5, (b) 0.5_15 and (c) 0.6_29 Cu-CHA catalysts at the end of the oxidation step, complementing Figure 4b-d in the main text, where the corresponding magnitudes of the FT-EXAFS spectra are reported. In each panel, the scaled components for Cu-species ❶, ❷/❷', ❸ are reported, vertically translated for the sake of clarity, together with percentages of each component over total Cu refined by ML-assisted EXAFS fitting. Please note how the lack of fit observed in the 2.25-2.75 Å (phase-uncorrected) range in the preliminary one-component EXAFS fits for 0.5_15 and 0.6_29 (see **Figure S10**) is substantially improved within the adopted three-component ML-assisted fitting model, mostly due to the contribution of component ❸, corresponding to 'bent' $[Cu_2(O_2)(NH_3)_4]^{2+}$ complexes with Cu-Cu interatomic distances of ca. 3 Å.

6 DR-UV-Vis spectra

The reactivity of $[Cu^I(NH_3)_2]^+$ complexes with O_2 at 200 °C was followed by DR UV-Vis spectroscopy, according to the protocol described in Section 1.2. The results obtained on sample 0.5_15 have been already published.[4] This section will mainly deal with the new results obtained on samples 0.1_5 and 0.6_29, which are reported in **Figure S14**, left panels. The spectrum measured at 200 °C after pretreatment in O_2 on 0.1_5 (black curve, top panel) is similar to what already reported on the same sample by Negri *et al.* [23] and on samples with similar Si/Al ratio by Li *et al.*[24] This feature has been explained in terms of Z_2Cu^{II} at *2Al* sites in 6r. On the other hand, the spectrum measured under the same condition on 0.6_29 (bottom panel, left) closely reminds that of sample 0.5_15, supporting the interpretation of the so-called 'quadruplet' (complex absorption in the d-d region, between 22000 and 8000 cm^{-1}) to $Z[Cu^{II}OH]$ at *1Al* sites in 8r and structurally related multimeric moieties $Z_x[Cu^{II}{}_xO_y]$.[23, 24]

After exposure to NO/NH_3 (light blue curves) in both samples the bands related to the *d-d* transitions of Cu^{II} ions in various configurations are consumed, together with a shift to higher wavenumbers of the ligand-to-metal charge transfer (LMCT) transitions, in agreement with the formation of $[Cu^I(NH_3)_2]^+$ complexes.[4] The final states after reaction with O_2 of the $[Cu^I(NH_3)_2]^+$ complexes (red curves in left panels) are directly compared for the three samples in the right panel of **Figure S14**. The shape of the corresponding spectrum for sample 0.5_15 (blue curve) has been explained in terms of $[Cu_2(NH_3)_4O_2]^{2+}$ complexes,[4] and is strikingly similar to that of sample 0.6_29 (dark blue). In agreement with XAS data, the spectrum of 0.1_5 shows a main difference in the region between 35000 and 22000 cm^{-1}, which is sensitive to the structure of Cu-oxo species.[4, 23-25]

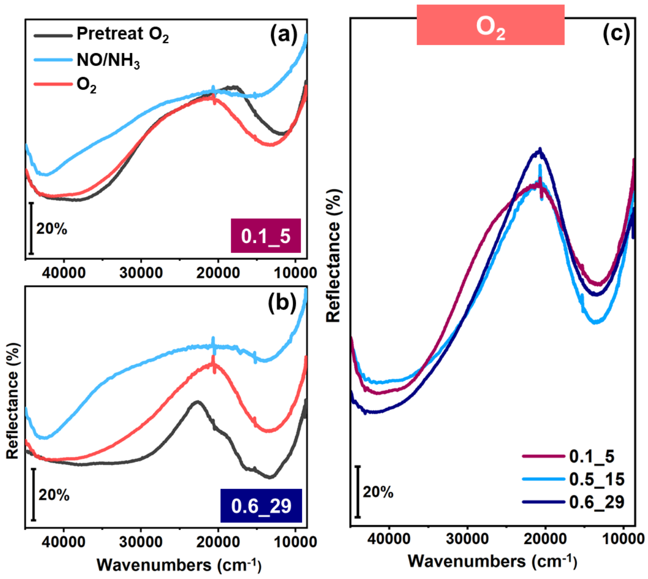

Figure S14: DR UV-Vis spectra for 0.1_5 (top, left) and 0.6_29 (bottom, left) Cu-CHA catalysts after pre-treatment in O_2, at the end of the reduction step and of the oxidation step. The right panel directly compares the three studied catalysts after the oxidation step.

7 References

(1) Janssens, T. V. W.; Falsig, H.; Lundegaard, L. F.; Vennestrøm, P. N. R.; Rasmussen, S. B.; Moses, P. G.; Giordanino, F.; Borfecchia, E.; Lomachenko, K. A.; Lamberti, C.; Bordiga, S.; Godiksen, A.; Mossin, S.; Beato, P., A Consistent Reaction Scheme for the Selective Catalytic Reduction of Nitrogen Oxides with Ammonia, *ACS Catal.*, **2015,** *5*, 2832-2845.

(2) Martini, A.; Borfecchia, E.; Lomachenko, K. A.; Pankin, I. A.; Negri, C.; Berlier, G.; Beato, P.; Falsig, H.; Bordiga, S.; Lamberti, C., Composition-Driven Cu-Speciation and Reducibility In Cu-CHA Zeolite Catalysts: A Multivariate XAS/FTIR Approach to Complexity, *Chem. Sci.*, **2017,** *8*, 6836-6851.

(3) Paolucci, C.; Parekh, A. A.; Khurana, I.; Di Iorio, J. R.; Li, H.; Albarracin Caballero, J. D.; Shih, A. J.; Anggara, T.; Delgass, W. N.; Miller, J. T.; Ribeiro, F. H.; Gounder, R.; Schneider, W. F., Catalysis in a Cage: Condition-Dependent Speciation and Dynamics of Exchanged Cu Cations in SSZ-13 Zeolites, *J. Am. Chem. Soc.*, **2016,** *138*, 6028-6048.

(4) Negri, C.; Selleri, T.; Borfecchia, E.; Martini, A.; Lomachenko, K. A.; Janssens, T. V. W.; Cutini, M.; Bordiga, S.; Berlier, G., Structure and Reactivity of Oxygen-Bridged Diamino Dicopper(II) Complexes in Cu-Ion-Exchanged Chabazite Catalyst for NH_3-Mediated Selective Catalytic Reduction, *J. Am. Chem. Soc.*, **2020,** *142*, 15884-15896.

(5) Negri, C.; Martini, A.; Deplano, G.; Lomachenko, K. A.; Janssens, T. V. W.; Borfecchia, E.; Berlier, G.; Bordiga, S., Investigating the Role of Cu-Oxo Species in Cu-Nitrate Formation over Cu-CHA Catalysts, *Phys. Chem. Chem. Phys.*, **2021,** *23*, 18322-18337.

(6) Mathon, O.; Beteva, A.; Borrel, J.; Bugnazet, D.; Gatla, S.; Hino, R.; Kantor, I.; Mairs, T.; Munoz, M.; Pasternak, S.; Perrin, F.; Pascarelli, S., The Time-Resolved and Extreme Conditions XAS (TEXAS) Facility at the European Synchrotron Radiation Facility: The General-Purpose EXAFS Bending-Magnet Beamline BM23, *J. Synchrotron Radiat.*, **2015,** *22*, 1548-1554.

(7) Bellet, D.; Gorges, B.; Dallery, A.; Bernard, P.; Pereiro, E.; Baruchel, J., A 1300 K Furnace for In Situ X-Ray Microtomography, *J. Appl. Crystallogr.*, **2003,** *36*, 366-367.

(8) Ravel, B.; Newville, M., ATHENA, ARTEMIS, HEPHAESTUS: Data Analysis for X-ray Absorption Spectroscopy Using IFEFFIT, *J. Synchrotron Radiat.*, **2005,** *12*, 537-541.

(9) Paolucci, C.; Khurana, I.; Parekh, A. A.; Li, S. C.; Shih, A. J.; Li, H.; Di Iorio, J. R.; Albarracin-Caballero, J. D.; Yezerets, A.; Miller, J. T.; Delgass, W. N.; Ribeiro, F. H.; Schneider, W. F.; Gounder, R., Dynamic Multinuclear Sites Formed by Mobilized Copper Ions in NOx Selective Catalytic Reduction, *Science*, **2017,** *357*, 898-903.

(10) Funke, H.; Scheinost, A. C.; Chukalina, M., Wavelet Analysis of Extended X-Ray Absorption Fine Structure Data, *Phys. Rev. B*, **2005,** *71*, 094110.

(11) Timoshenko, J.; Kuzmin, A., Wavelet Data Analysis of EXAFS Spectra, *Comput. Phys. Commun*, **2009,** *180*, 920-925.

(12) Martini, A.; Pankin, I. A.; Marsicano, A.; Lomachenko, K. A.; Borfecchia, E., Wavelet Analysis of a Cu-Oxo Zeolite EXAFS Simulated Spectrum, *Rad. Phys. Chem.*, **2020,** *175*, 108333.

(13) Pankin, I. A.; Martini, A.; Lomachenko, K. A.; Soldatov, A. V.; Bordiga, S.; Borfecchia, E., Identifying Cu-Oxo Species In Cu-Zeolites By XAS: A Theoretical Survey by DFT-Assisted XANES Simulation and EXAFS Wavelet Transform, *Catal. Today*, **2020,** *345*, 125-135.

(14) Martini, A.; Signorile, M.; Negri, C.; Kvande, K.; Lomachenko, K. A.; Svelle, S.; Beato, P.; Berlier, G.; Borfecchia, E.; Bordiga, S., EXAFS Wavelet Transform Analysis of Cu-MOR Zeolites for the Direct Methane to Methanol Conversion, *Phys. Chem. Chem. Phys.*, **2020,** *22*, 18950-18963.

(15) Penfold, T. J.; Tavernelli, I.; Milne, C. J.; Reinhard, M.; Nahhas, A. E.; Abela, R.; Rothlisberger, U.; Chergui, M., A Wavelet Analysis for the X-Ray Absorption Spectra of Molecules, *J. Chem. Phys.*, **2013,** *138*, 014104.

(16) Sushkevich, V. L.; Safonova, O. V.; Palagin, D.; Newton, M. A.; van Bokhoven, J. A., Structure of Copper Sites in Zeolites Examined by Fourier and Wavelet Transform Analysis of EXAFS, *Chem. Sci.*, **2020,** *11*, 5299-5312.

(17) Zabinsky, S. I.; Rehr, J. J.; Ankudinov, A.; Albers, R. C.; Eller, M. J., Multiple-Scattering Calculations of X-Ray-Absorption Spectra, *Phys. Rev. B*, **1995,** *52*, 2995-3009.

(18) Martini, A.; Bugaev, A. L.; Guda, S. A.; Guda, A. A.; Priola, E.; Borfecchia, E.; Smolders, S.; Janssens, K.; De Vos, D.; Soldatov, A. V., Revisiting the Extended X-ray Absorption Fine Structure Fitting Procedure through a Machine Learning-Based Approach, *J. Phys. Chem. A*, **2021,** *125*, 7080-7091.

(19) Martini, A.; Guda, S. A.; Guda, A. A.; Smolentsev, G.; Algasov, A.; Usoltsev, O.; Soldatov, M. A.; Bugaev, A.; Rusalev, Y.; Lamberti, C.; Soldatov, A. V., PyFitit: The Software for Quantitative Analysis of XANES Spectra Using Machine-Learning Algorithms, *Comput. Phys. Commun.*, **2020,** *250*, 107064.
(20) Joyner, R. W.; Martin, K. J.; Meehan, P., Some Applications of Statistical Tests in Analysis of EXAFS and SEXAFS Data, *J. Phys. C, Solid State Phys.*, **1987,** *20*, 4005-4012.
(21) Borfecchia, E.; Lomachenko, K. A.; Giordanino, F.; Falsig, H.; Beato, P.; Soldatov, A. V.; Bordiga, S.; Lamberti, C., Revisiting the Nature of Cu Sites in the Activated Cu-SSZ-13 Catalyst for SCR Reaction, *Chem. Sci.*, **2015,** *6*, 548-563.
(22) Pappas, D. K.; Borfecchia, E.; Dyballa, M.; Pankin, I. A.; Lomachenko, K. A.; Martini, A.; Signorile, M.; Teketel, S.; Arstad, B.; Berlier, G.; Lamberti, C.; Bordiga, S.; Olsbye, U.; Lillerud, K. P.; Svelle, S.; Beato, P., Methane to Methanol: Structure Activity Relationships for Cu-CHA, *J. Am. Chem. Soc.*, **2017,** *139*, 14961-14975.
(23) Negri, C.; Signorile, M.; Porcaro, N. G.; Borfecchia, E.; Berlier, G.; Janssens, T. V. W.; Bordiga, S., Dynamic Cu^{II}/Cu^{I} Speciation in Cu-CHA Catalysts by In Situ Diffuse Reflectance UV–Vis-NIR Spectroscopy, *Appl. Catal. A - Gen.*, **2019,** *578*, 1-9.
(24) Li, H.; Paolucci, C.; Khurana, I.; Wilcox, L. N.; Göltl, F.; Albarracin-Caballero, J. D.; Shih, A. J.; Ribeiro, F. H.; Gounder, R.; Schneider, W. F., Consequences of Exchange-Site Heterogeneity and Dynamics on the UV-Visible Spectrum of Cu-Exchanged SSZ-13, *Chem. Sci.*, **2019,** *10*, 2373-2384.
(25) Giordanino, F.; Vennestrom, P. N. R.; Lundegaard, L. F.; Stappen, F. N.; Mossin, S. L.; Beato, P.; Bordiga, S.; Lamberti, C., Characterization of Cu-Exchanged SSZ-13: A Compared FTIR, UV-Vis and EPR Study with Cu-ZSM-5 and Cu-β with Similar Si/Al and Cu/Al Ratios, *Dalton Trans.*, **2013,** *42*, 12741-12761.
